# Volume and Structure of Maxillofacial Trauma Before, During, and After COVID-19 Restrictions: A Multicentre Interrupted Time-Series and Period-Comparative Analysis of 3954 Patients

**DOI:** 10.3390/medicina62091628

**Published:** 2026-08-25

**Authors:** Ladislav Czako, Maria Janickova, Branislav Borza, Sarah Kalmanova, Rastislav Juricek, Marek Sovis, Anna Kobyliakova, Peter Kizek

**Affiliations:** 1 Department of Oral and Maxillofacial Surgery, University Hospital Bratislava Ruzinov, Faculty of Medicine, Comenius University, Ruzinovska 6, 826 06 Bratislava, Slovakia; 2 Department of Stomatology and Maxillofacial Surgery, University Hospital Martin, Jessenius Faculty of Medicine, Comenius University, Kollarova 2, 036 59 Martin, Slovakia; 3 Department of Stomatology and Maxillofacial Surgery, Louis Pasteur University Hospital Kosice, Faculty of Medicine, Pavol Jozef Safarik University, Rastislavova 43, 041 90 Kosice, Slovakia

**Keywords:** COVID-19, maxillofacial trauma, interrupted time-series, segmented regression, facial fractures, midface, mandible, micromobility, trauma epidemiology

## Abstract

*Background and Objectives:* COVID-19 restrictions altered population mobility and healthcare use. Most reports describe changes in facial-trauma counts; fewer characterise how injury composition changed or separate policy-associated changes from the secular trend. We examined the volume and structure of maxillofacial trauma around the March 2020 restrictions and the January 2022 easing of restrictions. *Materials and Methods:* A retrospective study of 3954 patients treated for confirmed facial fractures between 2018 and 2023 at three Slovak tertiary centres. The primary outcome was the monthly count of treated cases (case volume, not incidence, as no catchment denominator was available), modelled by segmented negative binomial regression with level and slope terms at two a priori interruptions and harmonic seasonality. Centre–month panel, centre-specific, and leave-one-centre-out models addressed between-centre heterogeneity. Structural outcomes were modelled by segmented binomial regression at the patient level, adjusting for centre, mechanism, sex, and age. *Results:* The restrictions were followed by a non-significant reduction in monthly case volume (count ratio 0.84, 95% confidence interval CI 0.67–1.04), and the easing was followed by an increase in the pooled series (1.50, 95% CI 1.22–1.86). This increase was strongly heterogeneous between centres (*p* < 0.001), was confined to one centre (1.86, 95% CI 1.42–2.43), and was compatible with no change once that centre was excluded (1.15, 95% CI 0.88–1.50). The non-mandibular fracture share fell at the onset of restrictions (odds ratio 0.54, 95% CI 0.38–0.77) and returned thereafter; the post-easing estimate was inconclusive. Electric-scooter injuries rose on a secular trend, with no level shift at either interruption. Conservative management increased at the easing and persisted after adjustment for case mix and centre (aOR 1.44, 95% CI 1.22–1.70). *Conclusions:* These periods were associated with changes in maxillofacial trauma composition, most consistently a shift toward conservative management. The post-easing volume rise was centre-specific, not a national rebound.

## 1. Introduction

The coronavirus disease 2019 (COVID-19) pandemic, declared by the World Health Organization in March 2020, was followed by wide-ranging restrictions on movement, the closure of public spaces, and the redirection of healthcare resources [1,2,3]. These measures coincided with changes in the epidemiology of injuries, and maxillofacial trauma—arising mainly from falls, interpersonal violence, road traffic, and sport—was among the areas affected [4,5].

A large international literature has recorded the crude changes that accompanied this period: reductions in traffic- and sport-related facial injuries during restrictions, increases in the relative share of fall-related trauma with a corresponding fall in interpersonal violence, and, in several settings, a shift toward conservative management [6,7,8,9]. Social distancing coincided with reduced overall trauma presentation [6], and the first months of the pandemic showed a distinct injury signature [7]. In parallel, and independently of the pandemic, the spread of shared electric scooters has been accompanied by a documented burden of craniofacial injury, both in its characteristic pattern and in the numbers presenting after such schemes were introduced [10,11].

Two gaps prompt the present work. First, most reports rely on before-and-after count comparisons that cannot separate a policy-associated change from the pre-existing secular trend or seasonality. Interrupted time-series (ITS) with segmented regression is the recommended quasi-experimental method for evaluating a population-level intervention with a defined start [12,13]; applied to oral and maxillofacial trauma, it remains uncommon [9]. Second, prior work has focused on how many patients presented rather than on how the composition of injury changed—the anatomical distribution of fractures, the age and sex profiles, the coupling between mechanisms and places of injury, and the management chosen. These features determine surgical workload and prevention targets more directly than headline case counts.

We therefore analysed a six-year, three-centre cohort using segmented regression together with multivariable modelling, focusing on the changing composition of maxillofacial trauma alongside its volume. We emphasise at the outset that this is an uncontrolled observational design without a non-exposed comparison series; it can identify changes that coincided in time with the two policy interruptions, but it cannot establish that the restrictions caused them.

### Objectives

We addressed the following overarching questions: Did the volume and the composition of maxillofacial trauma change around the introduction and relaxation of COVID-19 restrictions? Are any such changes distinguishable from the underlying secular trend, seasonality, and differences between the contributing centres?

The specific objectives were as follows. (1) To estimate immediate (level) and gradual (slope) changes in the monthly count of treated cases for the March 2020 restrictions and the January 2022 easing, and to test whether these estimates are consistent across the three centres. (2) To model the distribution of fracture sites over calendar time and to identify its independent predictors. (3) To describe age- and sex-specific changes and to distinguish secular from period-associated components of micromobility injury. (4) To map mechanism-by-location coupling and to model changes in the timing and modality of care.

## 2. Materials and Methods

### 2.1. Study Design and Setting

This was a retrospective multicentre observational study combining a segmented ITS analysis of the monthly count of treated cases with period-comparative analyses of injury composition from 1 January 2018 to 31 December 2023. Consecutive patients were identified from the maxillofacial trauma records of three university-based tertiary referral centres in Slovakia: University Hospital Bratislava Ruzinov (Faculty of Medicine, Comenius University), University Hospital Martin (Jessenius Faculty of Medicine, Comenius University), and Louis Pasteur University Hospital Kosice (Faculty of Medicine, Pavol Jozef Safarik University). Reporting followed the STROBE recommendations for observational studies [14] and the ITS reporting principles of Lopez Bernal and colleagues [12]; the specification and diagnostic reporting were guided by published reviews of methodological practice in ITS studies, which documented frequent under-reporting of autocorrelation and model diagnostics [15]. The completed STROBE checklist is provided as Supplementary File S1.

All three centres are the sole providers of specialist maxillofacial trauma care for their respective referral regions and operated continuously, with an uninterrupted 24-h emergency service, throughout the whole 72-month observation window, including during the periods of maximal COVID-19 hospital pressure. No centre suspended, merged, or transferred its maxillofacial trauma service, and admission criteria for facial fractures (radiographically or clinically confirmed fractures requiring specialist assessment) were unchanged. We are not aware of any formal changes in referral policy or catchment boundaries during the study window. We note, however, that we cannot exclude gradual, undocumented changes in local referral behaviour or in the completeness of departmental record-keeping, particularly at the Kosice centre, whose recorded caseload declined markedly across the first two study years (see Section 3.2); this possibility is addressed directly in the centre-stratified analyses and in the Strengths and Limitations section.

### 2.2. Participants

Patients were eligible if they had a radiographically or clinically confirmed fracture of the facial skeleton (mandible, midface, or upper facial third) treated at a participating centre during the study window, and if the core study variables were retrievable from the clinical record. Patients with facial soft-tissue injury without fracture, with isolated dentoalveolar injury, or with injuries outside the maxillofacial region were not eligible. Accordingly, every included patient had a facial fracture, and the anatomical categories in Section 2.4 therefore account for the whole analysed cohort.

Case identification and the absence of screening counts. Cases were identified by manual review of the maxillofacial trauma records held at each participating centre, and records were transcribed into the study database only where the study diagnoses were met. Presentations that did not meet those diagnoses were not transcribed or enumerated at the time of data collection because they were not relevant to the research question as it was then framed. Consequently, the number of records screened and the reasons for exclusion before cohort entry were not retrievable from the study’s database and could be reconstructed only by a fresh manual audit of the six years of trauma records at the three centres. The flow diagram (Figure S2) therefore begins at the point from which auditable counts existed, namely the 3954 included patients, and documents the onward flow into each analysis. The implications of this for selection bias are addressed in the Strengths and Limitations section.

### 2.3. Exposure and Interruptions

The exposure was continuous calendar time, expressed as sequential months (1–72). Two interruption points were specified a priori on the basis of national policy.

The first was in March 2020. An extraordinary situation was declared for the whole territory of Slovakia by Government Resolution No. 111 of 11 March 2020, effective from 06:00 on 12 March 2020. A state of emergency for healthcare providers was declared by Government Resolution No. 114 of 15 March 2020, effective from 16 March 2020 and subsequently extended by Resolutions No. 115 of 18 March 2020, No. 169 of 27 March 2020, No. 207 of 6 April 2020 and No. 233 of 16 April 2020. The decisive population-level restrictions were imposed by two measures of the Public Health Authority of the Slovak Republic: Measure No. OLP/2576/2020 of 12 March 2020, which closed specified premises and services, and Measure No. OLP/2595/2020 of 15 March 2020, which, from 06:00 on 16 March 2020, closed all retail outlets and service premises other than food shops, pharmacies, banks, post offices, and fuel stations. School closure and the postponement of non-urgent hospital activity followed in the same week [16,17,18,19]. The decisive national restrictions therefore took effect on 16 March 2020, that is, part-way through that month. For readers wishing to place these measures in a cross-national context, the Oxford COVID-19 Government Response Tracker provides an independently maintained record of containment and closure policies, coded on daily ordinal scales for more than 180 countries, including Slovakia, from 1 January 2020 onwards [20].

The second was in January 2022. Relaxation in Slovakia was staged rather than instantaneous: capacity and access rules were first relaxed on 19 January 2022, further exemptions for mass events followed on 26 February 2022, and remaining capacity and opening-hour restrictions on premises and events were withdrawn by 14 March 2022, with entry restrictions ending on 6 April 2022 [21,22]. January 2022 was chosen as the point at which the sequence of relaxations began. Because the easing was staged, this single calendar point is necessarily an approximation, and we therefore treat the placement of both interruptions as an explicit source of uncertainty and report a sensitivity analysis across a grid of alternative breakpoints (Section 2.6).

Partial-month exposure. The date of injury was recorded to the month and year, not the day; hence, exposure could not be apportioned within a month. Both interruption months were therefore classified as exposed in full (March 2020 and January 2022 were the first months of the respective segments); we now state plainly that this introduced a non-trivial amount of exposure misclassification rather than a negligible one. The restrictions took effect on 16 March 2020; 15 of the 31 days of March 2020 (48.4%) preceded them, yet they were counted as exposed. The easing began on 19 January 2022; 18 of the 31 days of January 2022 (58.1%) preceded it. In each interruption month, roughly half of the days are therefore misclassified, which will tend to bias the corresponding level-change estimates toward the null. Because injury dates are available only to the month, no finer allocation is possible with these data, and the ±2-month breakpoint grid (Section 2.6) was retained partly to quantify the consequences: shifting each interruption by one month in either direction brackets the true boundary, and the level-change estimates remained materially unchanged across that grid (Section 3.2).

Descriptive periods. The three descriptive periods used for the period-comparative analyses correspond exactly to the modelled interruptions: pre-restriction (1 January 2018–29 February 2020, 26 months), restriction (1 March 2020–31 December 2021, 22 months), and post-easing (1 January 2022–31 December 2023, 24 months). This differs from a calendar-year definition (2018–2019/2020–2021/2022–2023), under which 79 patients treated in January and February 2020 would be misassigned to the restriction period, despite having preceded it; the aligned definition is used for all descriptive results. Because the three periods are of unequal length, period totals are accompanied by mean monthly counts wherever volume is discussed.

### 2.4. Outcomes and Covariates

**Primary outcome.** The monthly number of patients treated for facial fractures across the three centres. No catchment population, person-time denominator, or offset was available, and the centres’ referral regions could not be delineated with the precision required to construct one. This outcome therefore measures hospital case volume, not incidence in the epidemiological sense, and we avoid the language of incidence throughout: model coefficients are reported as count ratios (CRs), that is, ratios of expected monthly counts, with 95% confidence intervals.

**Fracture site.** Each record listed every fractured anatomical region. Sites were grouped as *mandible* and *non-mandibular (midface and upper facial third)*, the latter comprising the zygomaticomaxillary complex, maxilla, orbit, blow-out and blow-in orbital fracture, naso-orbito-ethmoid complex, nasal bone, frontal bone, and alveolar process. A record was classified as combined if, and only if, it contained at least one mandibular *and* at least one non-mandibular site; records confined to one of the two groups were classified as isolated, irrespective of how many individual bones within that group were involved. Two coding decisions are stated explicitly because they are not self-evident. First, the frontal bone belongs to the upper facial third rather than the midface proper; it was grouped with the non-mandibular category for the primary analysis, and a sensitivity analysis excluding frontal-bone-only fractures is reported. Second, six records (0.2%) described a fracture region that could not be assigned to either group and were excluded only from the fracture-site models.

**Injury mechanisms and the primary-cause rule.** Mechanisms were recorded as fall, interpersonal violence, bicycle, electric scooter, road traffic, sport, gunshot, or other. In 220 records (5.6%), more than one mechanism was documented—most often a fall from a bicycle or scooter, or a fall during an assault. A single primary mechanism was assigned using a pre-specified hierarchy reflecting the external energy source rather than the terminal event: interpersonal violence > road traffic > electric scooter > bicycle > fall > sport > other (gunshot injuries, *n* = 4, were grouped with *other*). Thus “bicycle + fall” was coded as bicycle, and “assault + fall” as interpersonal violence. Because this ordering is a judgement rather than a fact, all fracture-site models were refitted under an alternative hierarchy, placing the rider’s mode of transport above road traffic (electric scooter > bicycle > road traffic > violence > sport > fall > other); the results were unchanged (Section 3.3). Micromobility was defined a priori as bicycle plus electric scooter combined, but is reported only as a supplementary composite, since the two mechanisms follow different temporal patterns (Section 3.4); bicycle and electric scooter are analysed separately as the principal mechanism-level outcomes.

**Management.** Three mutually exclusive categories were used, assigned by the highest level of intervention received: conservative (no reduction procedure); closed reduction (reduction and fixation without surgical exposure of the fracture, performed as a procedure under anaesthesia); and open reduction with internal fixation (ORIF). Closed reduction is deliberately not pooled with ORIF into a single “surgical” category, since the two differ in operative burden, anaesthetic requirement, and theatre time. The principal management outcome is therefore *conservative* versus *any reduction procedure*, with ORIF reported separately. This three-level terminology is used consistently in the Abstract, Results, Tables, and Figures.

**Early outcome.** The clinical records documented healing complications during the treatment episode and at the first scheduled post-treatment review, which occurred within six weeks of definitive treatment at all three centres. Uncomplicated early healing was therefore defined operationally as the absence, at that review, of any of the four recorded complication categories: secondary (delayed) healing, infection (osteonecrosis, osteomyelitis, or soft-tissue infection), bleeding, and other. This is a short-horizon, in-treatment outcome; it is not a measure of long-term functional result, and no standardised follow-up beyond the first review was available. Longer-term sequelae recorded at discharge from follow-up (hypoaesthesia/anaesthesia, paraesthesia, persistent pain, temporomandibular dysfunction, diplopia, plate exposure, facial asymmetry, malocclusion) were reported separately and descriptively.

**Covariates.** Age band, sex, alcohol or psychoactive-substance involvement, associated injuries, place of injury, time from injury to definitive care, and treating centre. Age was recorded only in bands (10–17, 18–29, 30–39, 40–49, 50–59, 60–69, ≥70); a continuous variable was therefore not available in the source data. For the regression models, age was approximated by band midpoints and entered continuously, per 10 years, with a quadratic term to allow curvature; band indicators were used for description. Two age thresholds appear in this manuscript, and their roles differ: ≥60 years is used as a covariate in patient-level models because it is the conventional threshold for the fall-dominant injury pattern in facial trauma, whereas ≥70 years is used descriptively as the oldest recorded band, since the source data offer no finer resolution above that point. No threshold was selected after inspecting outcomes.

### 2.5. Missing Data

Missingness was assessed variable by variable, by centre and by period (Table S3). This section concerns missing values *within* the included cohort; the separate question of records not captured before cohort entry is addressed in Section 2.2 and in the Strengths and Limitations section. The dataset was complete for all analysis variables, including the treating centre, and no analyses reported requiring the exclusion of any patients due to missing data. The only model fitted on fewer than the full cohort was the fracture-site model, which omitted six records (0.2%) whose fracture region could not be assigned to either anatomical group (*n* = 3948 of 3954, 99.8%). One non-analysis variable—operating surgeon’s age band—was missing in 1908 records (48.3%)—this is by design, since it is recorded only for operatively managed cases—and was not used in any analysis. Separately, we draw attention to an *informative* category that is not missingness in the statistical sense: alcohol or substance involvement was recorded as “not determined” in 3146 records (79.6%), with comparable proportions across periods (79.8%, 83.1%, 77.3%). Alcohol-related findings should therefore be read as documented positive exposure against an incompletely ascertained background, and we have qualified them accordingly.

### 2.6. Statistical Analysis

**Principal time-series model.** Categorical variables are summarised as counts and percentages. The monthly count of treated cases was modelled by segmented regression with a continuous time term, a level and a slope term for each of the two interruptions, and a harmonic pair (sine and cosine, period 12 months) for seasonality [12,13]:

log E(*Y_t_*) = β_0_ + β_1_·time*_t_* + β_2_·restrict*_t_* + β_3_·(time × restrict)*_t_* + β_4_·ease*_t_* + β_5_·(time × ease)*_t_* + β_6_·sin*_t_* + β_7_·cos*_t_*

Here, *Y_t_* is the count in month *t*; ‘restrict’ and ‘ease’ are indicators for the months from March 2020 and January 2022 onward; β_2_ and β_4_ are the immediate level changes; and β_3_ and β_5_ are the post-interruption slope changes.

The principal specification is negative binomial (NB2) rather than Poisson with HC1 standard errors. The Pearson dispersion statistic of the corresponding Poisson model was 1.76, confirming overdispersion; the estimated NB dispersion parameter was α = 0.0119. The Poisson-HC1 model is reported only as one of several sensitivity analyses, and complete coefficient sets for all models are given in Table S1.

**Seasonality specification.** Seasonality in time-series regression of count outcomes may be represented in several ways, including harmonic (Fourier) terms, calendar-period indicators, and flexible functions of time, each with different strengths [23]. Rather than assume a single harmonic pair, we therefore compared five seasonality specifications by AIC and BIC: no seasonality, one, two, and three harmonic pairs, and 11 calendar-month indicators (Table S4). The single harmonic pair was clearly preferred (AIC 542.0 versus 597.5 with no seasonality, 545.4 with two pairs, 547.7 with three pairs, and 550.8 with month indicators) and therefore retained.

**Autocorrelation and diagnostics.** Residual autocorrelation was assessed by the Durbin–Watson statistic, the Ljung–Box test at lags 6, 12 and 18, and inspection of the autocorrelation and partial autocorrelation functions (Figure S1). Because autocorrelation persisted after seasonal adjustment, four sensitivity specifications were fitted: a negative binomial model with Newey–West (HAC, 6 lags) standard errors; a model adding the log of the previous month’s count; a generalised least-squares model with a first-order autoregressive error on the log count (Cochrane–Orcutt); and the two-harmonic model. The lagged-count model deserves a caution that we now state explicitly in the Results: conditioning on the previous month’s count changes the estimand from a marginal to a conditional (dynamic) one, and because the lagged outcome lies on the causal path between an interruption and later months, part of the interruption effect is absorbed by the lag term. Its coefficients are therefore expected to be attenuated and are reported as a lower bound rather than as the preferred estimate. A full seasonal ARIMA model was not fitted: with 72 monthly observations and a count outcome, the level and slope terms of the segmented model remain more directly interpretable, and conclusions were consistent across all specifications tested.

**Between-centre heterogeneity.** Because the three centres contributed unequally and their shares changed across periods, four complementary analyses were performed. (i) A centre–month panel (3 centres × 72 months = 216 observations) was fitted with centre fixed effects and cluster-robust variance by centre. (ii) Centre-specific segmented models were fitted separately for each centre. (iii) Leave-one-centre-out models refitted the pooled series with each centre removed in turn. (iv) Centre-by-interruption heterogeneity was formally tested by a likelihood-ratio test comparing the panel model with and without centre × restriction and centre × easing interaction terms.

**Breakpoint sensitivity.** The analysis was repeated across a grid of 25 models, shifting each interruption point by up to two months in either direction; the ranges of level-change estimates and *p*-values were reported in full.

**Figure 1:**
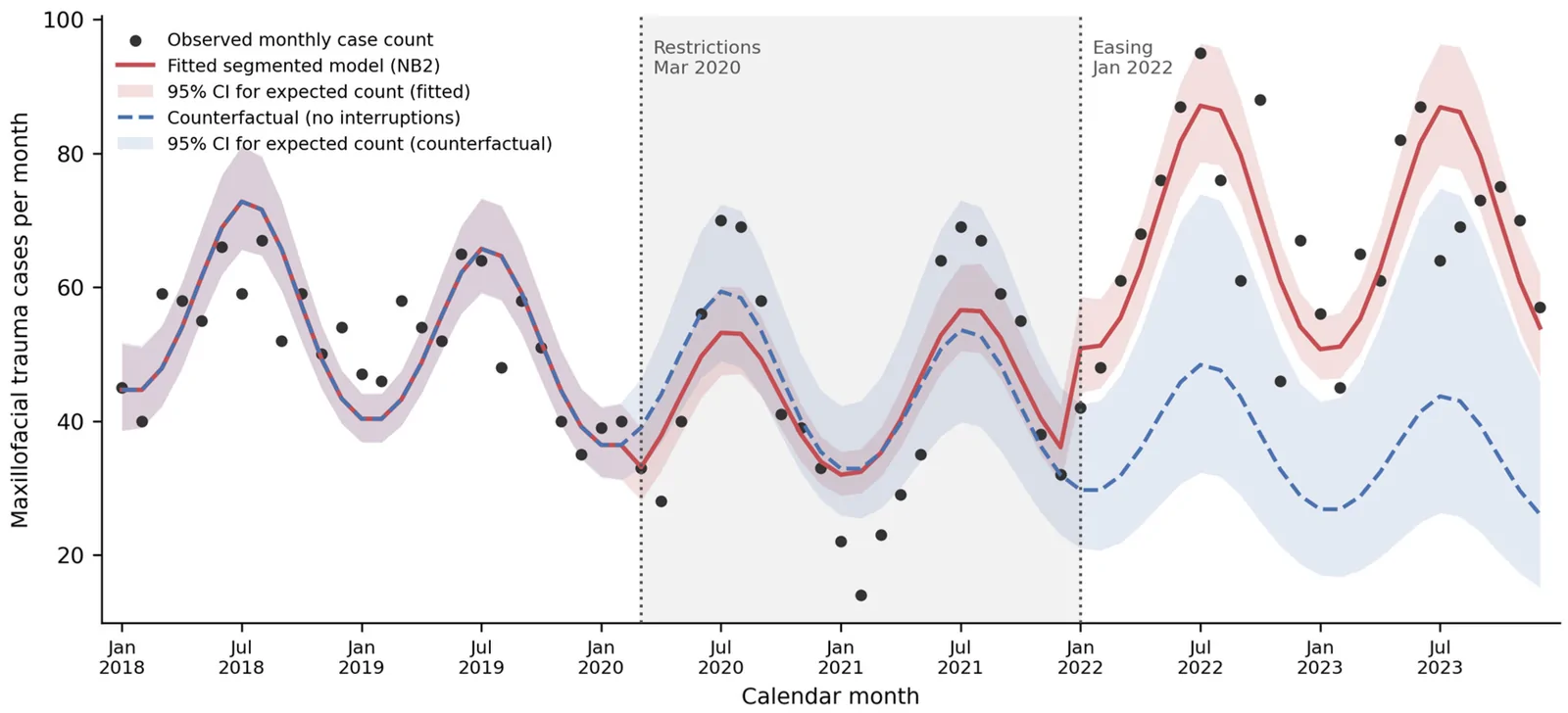
Segmented interrupted time-series of the monthly count of treated maxillofacial trauma cases, 2018–2023. Points are observed monthly counts; the solid line is the fitted segmented negative binomial model, and the dashed line is the counterfactual expected in the absence of the two interruptions. Shaded bands are 95% confidence intervals for the expected monthly count, derived from the sampling uncertainty of the model coefficients alone; they do not incorporate the dispersion of the negative binomial observation process and are therefore not prediction intervals for an individual future month, which would be wider.

**Figure 2:**
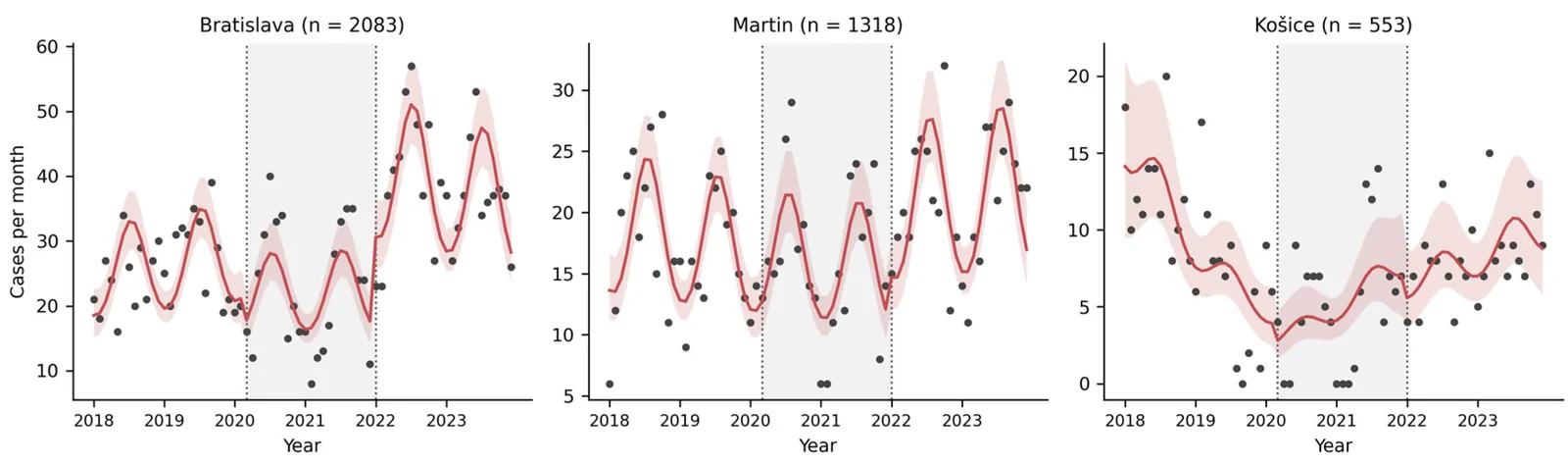
Centre-specific segmented interrupted time-series. Shaded bands are 95% confidence intervals for the expected monthly count (coefficient uncertainty only; see Figure 1). Post-easing level increases are present in Bratislava, borderline in Martin, and absent in Kosice.

**Figure 3:**
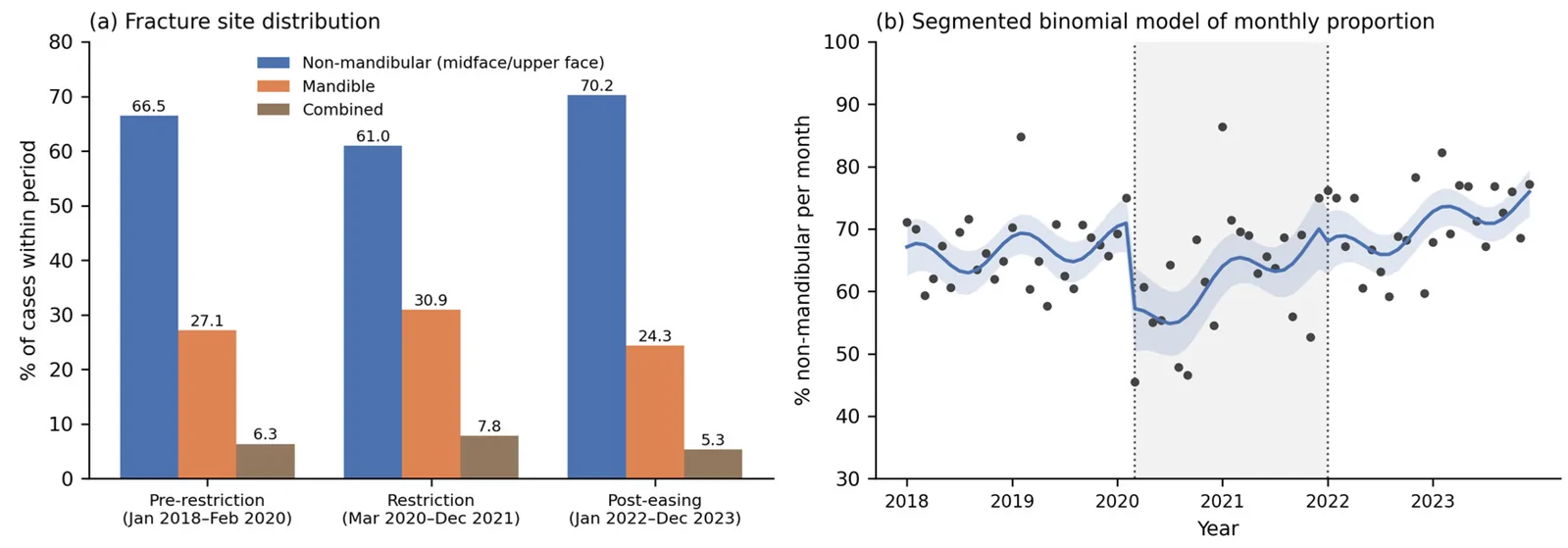
Fracture sites. (**a**) Distribution within each period as percentages of cases within the period. (**b**) Segmented binomial model of the monthly proportion of non-mandibular fractures; the shaded band is the 95% confidence interval for the expected proportion (coefficient uncertainty only; see Figure 1).

**Counterfactual and cumulative excess.** The counterfactuals were generated by setting all four interruption terms to zero and predicting expected counts for the post-easing months. The cumulative excess was computed as 100 × (Σ*Y*_obs_ − Σ*Y*_cf_)/Σ*Y*_cf_ over 49–72 months. Uncertainty was obtained by Monte Carlo simulation: 10000 coefficient vectors were drawn from the multivariate normal distribution defined by the fitted coefficients and their covariance matrix, the counterfactual total was recomputed for each draw, and the 2.5th and 97.5th percentiles of the resulting distribution were reported. We emphasise what this procedure captures and what it does not. It propagates only the sampling uncertainty of the estimated coefficients; it does not incorporate the additional dispersion of the negative binomial observation process. The resulting quantities are therefore 95% confidence intervals for the expected (mean) count under the model, rather than prediction intervals for a future observed count, which would be appreciably wider. They are labelled accordingly throughout this manuscript, including in the bands shown in Figure 1, Figure 2 and Figure 3, and the same qualification applies to the interval reported for the cumulative excess. The excess was recomputed under alternative pre-trend and seasonality specifications and under centre-specific models.

**Structural outcomes over calendar time.** The key compositional outcomes are not assessed by cross-period χ^2^ alone, since such comparisons ignore secular trend, seasonality, and the changing size and composition of the caseload. For each of the monthly proportions—non-mandibular fracture, bicycle injury, electric-scooter injury, interpersonal violence, road traffic, sport, patients aged ≥60 and ≥70 years, female sex, conservative management, ORIF, same-day definitive care, alcohol involvement, and early complications—a segmented binomial regression was fitted with the same time, level, slope, and harmonic structure as the principal model and HC1 standard errors (Table S5).

**Patient-level models.** The fracture site was modelled with a multinomial logistic regression, retaining all three categories (non-mandibular as the reference, versus mandible and versus combined); hence, combined injuries were no longer discarded. The covariates were the period, mechanism, sex, age (continuous, per 10 years, plus the quadratic term), and treating centre. A binary logistic model—restricted to isolated fractures—is reported alongside it for comparability with the previous literature; for that model, we report the *c*-statistic, the Hosmer–Lemeshow test, complete-case counts, and a formal test of the period × mechanism interaction. Management (conservative versus any reduction procedure) was modelled by logistic regression adjusting for the mechanism, fracture site, number of fracture sites, associated injury, sex, age, and centre.

**Multiplicity.** The segmented time-series model of monthly case volume was the single primary analysis. All period-comparative and structural comparisons were pre-specified secondary analyses and are interpreted as such; the family of secondary period comparisons was controlled with the Benjamini–Hochberg false-discovery-rate procedure [24], and both *p* and *q* values are reported (Table S2). Tests were two-sided with significance at *p* < 0.05. Analyses were conducted in Python 3.12 (statsmodels 0.14, SciPy 1.11) and cross-checked in IBM SPSS Statistics for Windows, version 28.0 (IBM Corp., Armonk, NY, USA).

**Interpretation.** Because the design has no non-exposed control series, all estimates are reported in associative language. Level and slope terms are described as changes *coinciding with* or *following* an interruption, not as effects caused by it.

### 2.7. Ethics

The study was conducted in accordance with the Declaration of Helsinki. Ethical review and approval were waived by the institutional review boards of the three participating centres because the study was a retrospective analysis of a fully anonymised minimal dataset derived from routine clinical care: Ethics Committee of Jessenius Faculty of Medicine, Comenius University (waiver EC-MT-69/2026, 27 July 2026); Ethics Committee of University Hospital Bratislava Ruzinov/Faculty of Medicine, Comenius University (waiver EC-BA-56/2026, 18 August 2026); Ethics Committee of Louis Pasteur University Hospital Kosice/Faculty of Medicine, Pavol Jozef Safarik University (waiver EC-KE-08071/2026, 14 August 2026). Patient consent was waived on the same grounds.

## 3. Results

### 3.1. Cohort

A total of 3954 patients met the inclusion criteria: 1361 (34.4%) in the pre-restriction period, 974 (24.6%) during restrictions, and 1619 (40.9%) after the easing. Because the periods differ in length (26, 22, and 24 months), the corresponding mean monthly counts are more informative: 52.3, 44.3, and 67.5 cases per month. Men accounted for 2990 cases (75.6%) and women for 964 (24.4%). Full demographic and clinical characteristics by period are given in Table 1, and the distribution by centre in Table 2 (Bratislava 2083, Martin 1318, Kosice 553). Approximate mean age (from band midpoints) was 42.0, 42.4, and 43.5 years across the three periods (Kruskal–Wallis, *p* = 0.078).

Relative to the pre-restriction period, total volume was 28.4% lower during restrictions and 19.0% higher afterwards; in mean monthly terms, the corresponding changes were −15.4% and +28.9%.

### 3.2. Monthly Case Volume: Segmented Time-Series

The principal negative binomial segmented model is shown in Figure 1, with the complete coefficient set in Table 3 and Table S1. Against a small and non-significant underlying downward trend (CR per month 0.992, 95% CI 0.982–1.001; *p* = 0.077), the March 2020 restrictions were followed by a non-significant immediate reduction in the level of the series (CR 0.84, 95% CI 0.67–1.04; *p* = 0.111) and a non-significant change in slope (CR 1.014 per month, 95% CI 0.998–1.030; *p* = 0.083). The January 2022 easing was followed by an upward level shift in the pooled series (CR 1.50, 95% CI 1.22–1.86; *p* < 0.001), with no accompanying slope changes (CR 0.995 per month, 95% CI 0.979–1.010; *p* = 0.50). Seasonality was strong, with summer peaks (for both harmonic terms, *p* < 0.001).

**Overdispersion, autocorrelation, and model diagnostics.** The Pearson dispersion statistic of the Poisson model was 1.76, and the negative binomial dispersion parameter was α = 0.0119, supporting the negative binomial as the principal specification. Residual autocorrelation persisted after seasonal adjustment (Durbin–Watson 1.24; Ljung–Box, *p* < 0.001 at lags 6, 12, and 18; residual ACF at lag 1 = 0.38, above the 95% bound of 0.23), with the ACF and PACF shown in Figure S1. Estimates were nevertheless stable across specifications (Table S1): Poisson-HC1 gave an easing level shift of 1.48 (95% CI 1.20–1.84), Newey–West HAC standard errors 1.50 (1.19–1.91), a generalised least-squares model with an AR(1) error (ρ = 0.44) 1.52 (1.10–2.10; *p* = 0.013), and the two-harmonic model 1.52 (1.23–1.88). Adding the previous month’s log-count removed the residual autocorrelation (Ljung–Box *p* = 0.14) and attenuated the easing level shift to 1.32 (1.07–1.63; *p* = 0.011). As set out in Section 2.6, this dynamic specification conditions on an intermediate variable and its coefficient is interpreted as a lower bound rather than as the preferred estimate. Neither slope term reached significance in any specification.

**Breakpoint placement.** Across the 25 models, shifting each interruption by up to two months, the easing level shift ranged from 1.30 to 1.56 and remained significant in all models (maximum *p* = 0.020), while the restriction level shift was non-significant throughout (minimum *p* = 0.051). The pooled results are therefore not an artefact of the exact calendar month chosen, which is reassuring given the staged nature of the 2022 relaxation.

**Between-centre heterogeneity.** The centre composition of the cohort changed across periods: Bratislava contributed 49.2%, 51.1%, and 56.6% of cases in the three periods, while Kosice contributed 17.6%, 12.0%, and 12.2% (Table 2). This heterogeneity is material to the interpretation of the pooled series. A likelihood-ratio test of the centre × interruption interaction in the centre–month panel was highly significant (χ^2^ = 27.28, df = 4, *p* < 0.001), so the three centres did not move together.

Centre-specific models (Figure 2, Table 4) show why. The post-easing increase was confined to Bratislava (CR 1.86, 95% CI 1.42–2.43; *p* < 0.001); it was borderline at Martin (1.32, 95% CI 0.99–1.75; *p* = 0.058) and absent at Kosice (0.80, 95% CI 0.42–1.50; *p* = 0.48). Leave-one-centre-out analyses confirm the dependence: excluding Bratislava, the pooled easing estimate fell to 1.15 (95% CI 0.88–1.50; *p* = 0.29), whereas excluding Kosice or Martin left it at 1.64 (1.34–2.01) and 1.59 (1.21–2.09), respectively. In the centre–month panel with centre fixed effects and cluster-robust variance, the easing estimate was 1.40 (95% CI 0.92–2.14; *p* = 0.12)—that is, compatible with no change—while the restriction level shift became nominally significant (0.84, 95% CI 0.71–0.99; *p* = 0.039). However, with only three clusters, the cluster-robust intervals are themselves imprecise and should be read cautiously.

The Kosice series (Figure 2) additionally shows a steep decline over 2018–2019, before any pandemic exposure, from a mean of about 14 to about 6 cases per month. A fall of this magnitude in the pre-interruption segment is more consistent with a change in referral behaviour or in the completeness of departmental record capture than with a change in injury occurrence, and it is precisely the kind of centre-level artefact that a pooled series can transmit into an apparent national trend.

**Cumulative excess relative to the counterfactual.** Over the 24 post-easing months, 1619 cases were observed against a modelled counterfactual of 863 cases (95% CI for the expected count 556–1375), with a cumulative excess of 87.7% (95% CI 17.7% to 191.2%). During the restriction period, the observed volume was close to the counterfactual (974 observed versus 985 expected; −1.1%, 95% CI −21.9% to +24.5%). As set out in Section 2.6, these intervals reflect coefficient uncertainty in the expected count and exclude the additional dispersion of the observation process.

Three qualifications materially limit what the 87.7% figure can support, and we now state them prominently. First, its confidence interval is very wide, spanning a tenfold range; the point estimate alone conveys a precision the data do not contain, and this is visible in the widening counterfactual band in Figure 1. Second, the estimate is highly sensitive to the assumed pre-interruption trend: it is 89.2% under the two harmonic pairs and 94.2% (95% SI 0.8–268.4%) with no seasonality term, but only **25.5%** if the modest pre-interruption downward trend is not extrapolated forward. Because that trend is estimated from only 26 pre-interruption months, projecting it across four further years is a strong assumption, and the discrepancy between the 19.0% observed rise in total volume and the 87.7% excess over the counterfactual is largely an artefact of that extrapolation. Third, the excess is concentrated in a single centre, as shown above. We therefore no longer describe the post-easing pattern as a real national rebound, and we regard the counterfactual excess as a model-dependent quantity to be interpreted alongside, not in place of, the observed change.

### 3.3. Fracture Site

The distribution of fracture sites differed across periods (χ^2^, *p* < 0.001; *q* < 0.001) (Figure 3a, Table 5). Non-mandibular fractures predominated throughout, but their share fell during restrictions (66.5% → 61.0%) and rose afterwards (70.2%), while isolated mandibular fractures moved in the opposite direction (27.1% → 30.9% → 24.3%), and combined fractures peaked during restrictions (6.3% → 7.8% → 5.3%). The ratio of non-mandibular to mandibular fractures thus contracted from 2.45 before to 1.97 during the restrictions, before widening to 2.89.

Modelling the monthly proportion over calendar time (segmented binomial regression) (Figure 3b, Table S5) localises this pattern in time. The proportion of non-mandibular fractures fell abruptly at the onset of restrictions (OR 0.54, 95% CI 0.38–0.77; *p* < 0.001) and then recovered gradually (slope changes OR 1.023 per month, 95% CI 0.998–1.048; *p* = 0.068). Critically, there were **no** level changes at the easing (OR 0.85, 95% CI 0.62–1.18; *p* = 0.34) and no post-easing slope changes (*p* = 0.45). The compositional signal is therefore a transient shift toward mandibular injury during the restriction period, followed by return—not a new post-easing state.

The multinomial model retaining all three categories (*n* = 3948; Table 6) gives the same picture and adds centre. Relative to non-mandibular fracture and to the pre-restriction period, the restriction period was associated with higher relative risks of both mandibular fracture (RRR 1.25, 95% CI 1.03–1.51; *p* = 0.023) and combined fracture (RRR 1.41, 95% CI 1.01–1.97; *p* = 0.042). Post-easing estimates were not significantly different for either mandibular (RRR 0.85, 95% CI 0.72–1.01; *p* = 0.068) or combined fractures (RRR 0.85, 95% CI 0.62–1.17; *p* = 0.33). Age was the strongest predictor of a non-mandibular pattern (RRR for mandible 0.71 per 10 years, 95% CI 0.67–0.75; *p* < 0.001, with a small quadratic term, *p* = 0.012), and mechanism was independently informative: relative to falls, bicycle (RRR 0.61), road-traffic (0.49), sport (0.44), and other (0.64) injuries were associated with non-mandibular fracture, while road traffic (RRR 2.85), bicycle (1.76), interpersonal violence (1.66) and other (2.16) mechanisms were associated with combined fracture. Treating centre was independently associated with fracture site (Kosice versus Bratislava, RRR for mandible 0.54, 95% CI 0.42–0.69; for combined fracture 1.85, 95% CI 1.33–2.57), underlining that centre must be modelled rather than assumed away.

The binary logistic model restricted to isolated fractures (*n* = 3700) was consistent (post-easing aOR for mandible 0.85, 95% CI 0.71–1.01; *p* = 0.066), with acceptable calibration (Hosmer–Lemeshow, *p* = 0.11) and modest discrimination (*c* = 0.670). Period × mechanism interaction was not supported (*p* = 0.98). Results were unchanged when frontal-bone-only fractures were excluded (post-easing aOR 0.85; *p* = 0.077; *n* = 3633) and when the alternative mechanism hierarchy was used (aOR 0.85; *p* = 0.067).

**We therefore interpret the post-easing fracture-site estimate as inconclusive.** This is a consistent but non-significant tendency, and it does not constitute evidence of an independent structural shift beyond case mix.

### 3.4. Injury Mechanism

**Figure 4:**
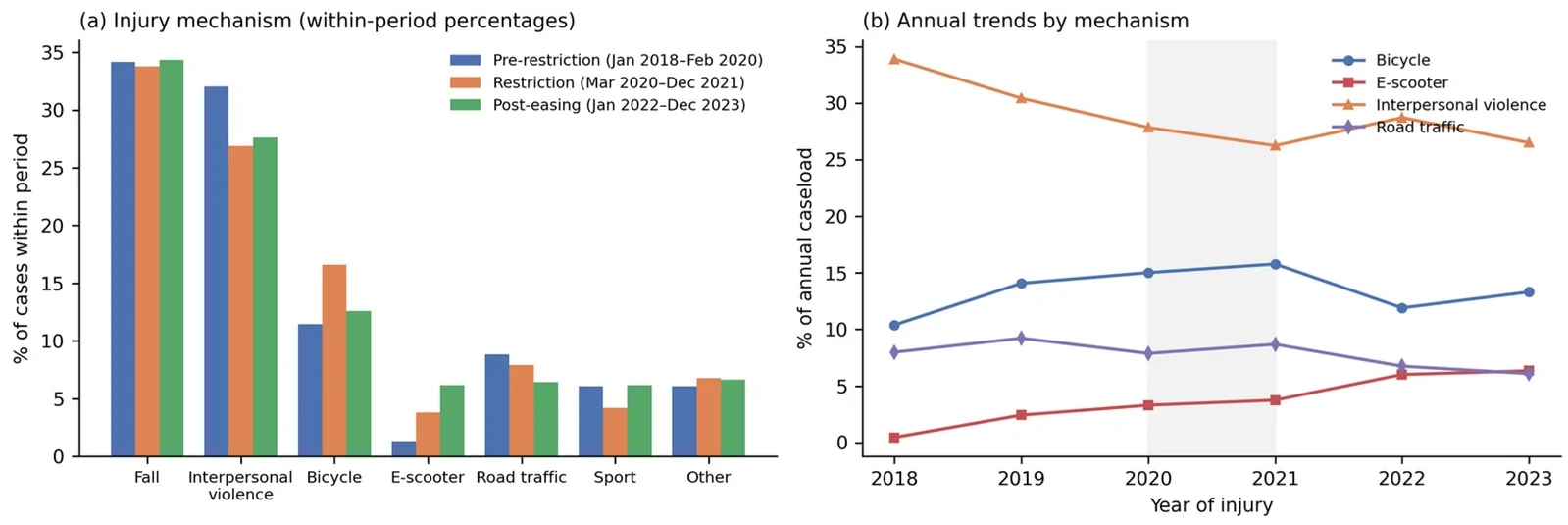
Injury mechanisms. (**a**) Within-period percentages by mechanisms. (**b**) Annual trends for selected mechanisms.

Mechanism distribution differed across periods (χ^2^, *p* < 0.001; *q* < 0.001) (Table 7, Figure 4a). Falls were the leading mechanism and were strikingly stable in share (34.2% → 33.8% → 34.3%). Interpersonal violence fell during restrictions and only partly recovered (32.0% → 26.9% → 27.6%). Road-traffic injuries declined across the study (8.8% → 7.9% → 6.4%), and sport dipped during restrictions before returning to baseline (6.1% → 4.2% → 6.2%).

Bicycle and electric-scooter injuries are analysed separately, and the distinction matters. Bicycle injuries rose during restrictions and then fell back toward their earlier share (11.5% → 16.6% → 12.6%), with no significant level or slope term in the segmented binomial model. Electric-scooter injuries, by contrast, increased monotonically (1.3% → 3.8% → 6.2%), and the segmented model attributes this to a **continuous secular trend rather than to either interruption**: the underlying monthly trend in the scooter share was strongly positive (OR 1.134 per month, 95% CI 1.052–1.221; *p* = 0.001), the slope *decelerated* during restrictions (OR 0.884, 95% CI 0.812–0.961; *p* = 0.004), and neither interruption produced a significant level shift (restriction *p* = 0.31; easing *p* = 0.12). The annual trajectories in Figure 4b show the same thing. The composite micromobility indicator (12.8% → 20.4% → 18.8%) conceals these two opposite patterns and is reported only for comparability with the earlier literature.

### 3.5. Demographic Composition

The sex distribution shifted across periods (χ^2^, *p* = 0.031; *q* = 0.045): the male-to-female ratio narrowed from 3.54 before to 2.76 during the restrictions, before partially recovering to 3.00. The segmented binomial model localises this to the restriction period (female share, level OR 1.53, 95% CI 1.08–2.17; *p* = 0.017), with no changes at the easing (*p* = 0.41)—consistent with the temporary reduction in male-predominant assault. The mechanism was strongly sex-patterned overall: interpersonal violence accounted for 34.9% of male but 10.5% of female trauma, whereas falls accounted for 56.0% of female versus 27.1% of male trauma.

The oldest age band (≥70 years) grew as a share of the cohort (9.5% → 9.4% → 12.4%; χ^2^ *p* = 0.015, *q* = 0.024). This period-level difference should, however, be treated with restraint: in the segmented binomial model neither interruption produced a significant level or slope change in the ≥70 share (all *p* > 0.16), and the underlying monthly trend was positive but not significant (OR 1.015, 95% CI 0.998–1.032; *p* = 0.092). Ageing of the injured population is therefore best described as a gradual drift over the study window rather than as a change tied to either policy point. Among patients aged ≥60 years, falls caused 69.5% of injuries, rising to 79.5% in women.

**Figure 5:**
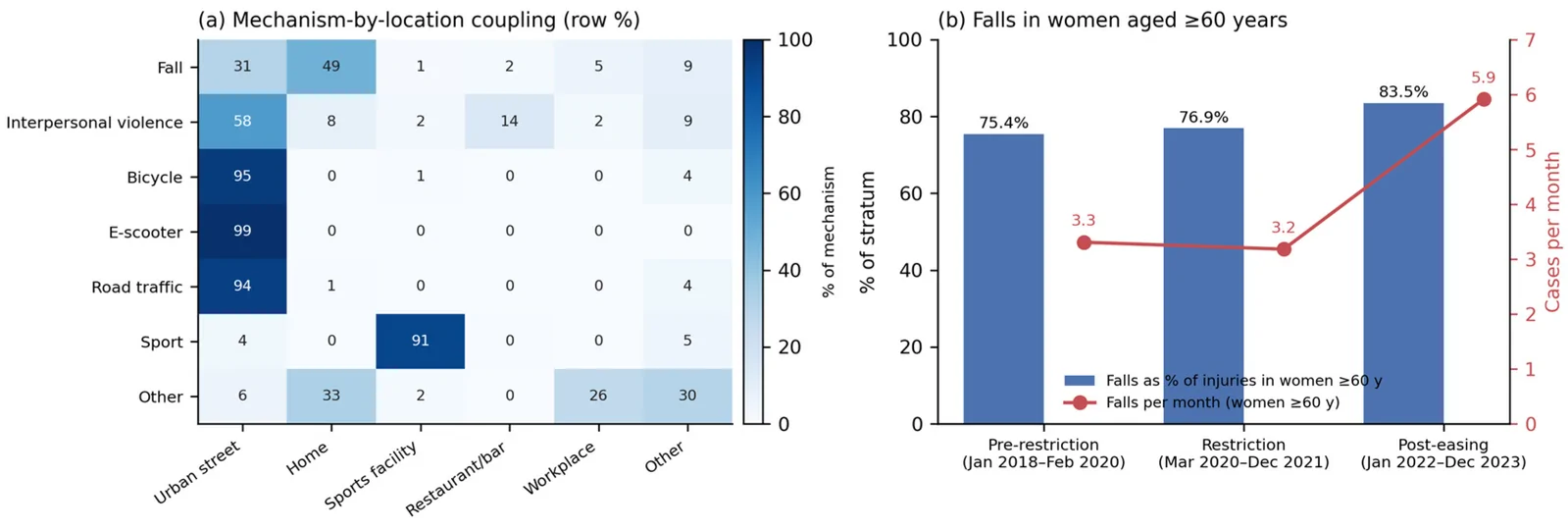
(**a**) Mechanism-by-location coupling: percentage of each mechanism occurring at each place of injury (row percentages). (**b**) Falls in women aged ≥60 years as a percentage of injuries within that stratum (bars) and as cases per month (line).

Falls in women aged ≥60 years are presented as proportions and monthly rates rather than as absolute counts (Figure 5b), since the periods differ in both length and total size. Falls made up 75.4%, 76.9%, and 83.5% of injuries in this stratum across the three periods, and the monthly rate was 3.3, 3.2, and 5.9 cases per month. The stratum itself also grew as a share of the cohort (8.4% → 9.3% → 10.5%). The post-easing increase in this group is therefore real in rate terms, but considerably more modest than a comparison of absolute counts across periods would suggest.

### 3.6. Place of Injury and Mechanism-by-Location Coupling

The place of injury differed across periods (χ^2^, *p* < 0.001; *q* < 0.001) (Table 8). Urban streets predominated throughout, dipping during restrictions (50.0% → 48.4%) and rising afterwards (54.9%). Home injuries were a stable one-fifth of cases (20.6% → 21.0% → 21.9%). The mechanism-by-location analysis (Figure 5a) shows that the home environment was almost exclusively a fall setting, and that home-based falls formed a near-constant 15.8–17.1% of all traumas in every period (655 cases in total; 9.0, 7.0, and 11.2 cases per month). The widely reported “shift to domestic injuries” therefore reflects a relative reduction in competing street-based mechanisms during restrictions rather than an absolute rise in domestic falls.

### 3.7. Management, Timing of Care, and Early Outcome

**Figure 6:**
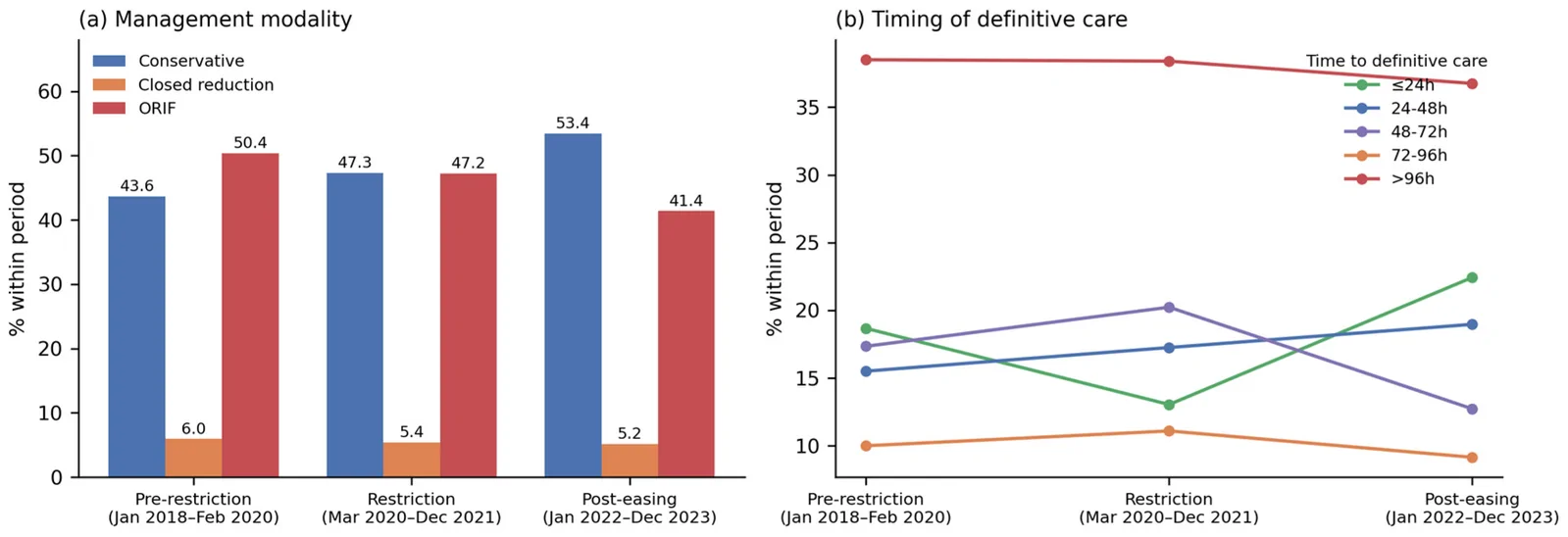
Management and timing of care. (**a**) Management modality as within-period percentages, with closed reduction shown separately from ORIF. (**b**) Distribution of time from injury to definitive care by periods.

Management differed across periods (χ^2^, *p* < 0.001; *q* < 0.001) (Table 9, Figure 6a). Conservative management rose progressively (43.6% → 47.3% → 53.4%), ORIF fell (50.4% → 47.2% → 41.4%), and closed reduction remained a small and stable category (6.0% → 5.4% → 5.2%).

This is the compositional change most robustly supported by the time-series modelling. In the segmented binomial model, the conservative share showed a significant upward level shift at the easing (OR 1.56, 95% CI 1.12–2.18; *p* = 0.009) with a corresponding fall in the ORIF share (OR 0.69, 95% CI 0.51–0.93; *p* = 0.014), on top of a modest underlying upward trend in conservative management (OR 1.023 per month, 95% CI 1.003–1.042; *p* = 0.023). It also survived patient-level adjustment: in a logistic model of conservative versus any reduction procedure (*n* = 3954), adjusting for the mechanism, fracture site, number of fracture sites, associated injury, sex, age, and centre, the post-easing period retained an independent association with conservative management (aOR 1.44, 95% CI 1.22–1.70; *p* < 0.001), while the restriction period did not (aOR 1.17, 95% CI 0.97–1.41; *p* = 0.10). Case mix behaved as expected (mandibular fracture aOR 0.34; combined 0.47; each additional fracture site 0.69), and centre was again a very strong predictor (Martin versus Bratislava aOR 2.46; Kosice 0.13), indicating substantial variation in local operative thresholds that is independent of period.

Timing of definitive care also changed (χ^2^, *p* < 0.001; *q* < 0.001): same-day (≤24 h) definitive treatment fell during restrictions (18.7% → 13.0%) and rose afterwards (22.4%), a pattern confirmed by segmented binomial modelling (restriction level OR 0.55, 95% CI 0.35–0.86, *p* = 0.008; easing level OR 1.97, 95% CI 1.29–2.99, *p* = 0.002). The proportion treated after 96 h was broadly stable (38.5% → 38.4% → 36.8%). Documented alcohol or substance involvement dipped during restrictions and then exceeded baseline (20.2% → 16.9% → 22.7%; *p* = 0.002, *q* = 0.004), although—as noted in Section 2.5—involvement was not determined in about 80% of records in any period; hence, this contrast rests on the documented positives only.

Uncomplicated early healing, as defined in Section 2.4, occurred in 94.0%, 95.1%, and 94.3% of patients across the three periods (*p* = 0.51). Recorded early complications were secondary healing (31, 17, 28 cases), infection (25, 9, 26), bleeding (8, 11, 17), and other (18, 11, 22). Longer-term sequelae recorded at the end of follow-up were documented in 22.5%, 25.2%, and 20.0% of patients (*p* = 0.009; *q* = 0.016), most commonly hypoaesthesia or anaesthesia and paraesthesia. Because follow-up was not standardised, its completeness cannot be verified from the available records, and the possibility of differential ascertainment across periods cannot be excluded. Therefore, these outcome data are presented descriptively; they are consistent with, but do not demonstrate, the preserved quality of acute care.

## 4. Discussion

This six-year, three-centre study describes the volume and the composition of maxillofacial trauma around two national policy interruptions. Its central message can be stated plainly: the pooled series show an increase in monthly case volume following the January 2022 easing, but this increase is not consistent across contributing centres, and the accompanying compositional changes are more modest, more time-localised, and, in one important case, entirely secular.

**Case volume.** The segmented model separates the secular trend and seasonality from the changes at the interruptions, and on that basis the restriction period was not associated with a significant reduction in the level of the pooled series, while the easing was followed by an upward level shift of about 50%. The centre-stratified analyses, however, show that this pooled estimate is not a general phenomenon: it is driven in the Bratislava centre, borderline in Martin, and absent in Kosice. The formal test of centre-by-interruption heterogeneity is highly significant, and the estimate becomes compatible with no change both when Bratislava is removed and when the centre fixed effects and cluster-robust variance are used. A pooled ITS based on aggregated multicentre counts can transmit changes in one centre’s referral patterns or record capture into what appears to be a system-wide signal, and the pronounced pre-pandemic decline in the Kosice series is a concrete illustration of that risk. Other multicentre series from this period, including tertiary-centre cohorts from Madrid and a French multicentre comparison, have likewise reported period-associated changes in facial-trauma volume and case mix [25,26]; our findings suggest that such pooled comparisons deserve centre-level scrutiny before they are read as regional or national effects. We therefore present the volume finding as a centre-dependent association rather than as evidence of a national rebound in facial trauma, and we would caution against using the pooled counterfactual excess for capacity planning without local corroboration.

The cumulative excess over the modelled counterfactual illustrates a more general point about ITS reporting. While an excess of 87.7% sounds decisive, its 95% confidence interval of 17.7–191.2% does not, and the same quantity falls to 25.5% if the pre-interruption trend—estimated from 26 months—is not extrapolated across the following four years. Reporting such an excess without an uncertainty interval and without sensitivity to the pre-trend assumption is likely to overstate what a short pre-interruption segment can support; we would encourage the routine reporting of both.

**Fracture site.** Modelling the monthly proportion over calendar time places the compositional signal firmly in the restriction period: the share of non-mandibular fractures fell sharply at the onset of restrictions and then recovered, with no level changes at the easing. This is mechanistically coherent—interpersonal violence, which more often fractures the mandible, made up a larger relative share, while street-based and sporting mechanisms were suppressed—and it is corroborated by the multinomial model, in which the restriction period was associated with both mandibular and combined fractures. The post-easing period, by contrast, showed only a consistent, non-significant tendency toward a non-mandibular pattern (aOR 0.85, *p* = 0.07) that was robust in direction across three sensitivity analyses but never reached significance. We interpret it as inconclusive and draw no conclusion about an independent post-easing structural shift. If a genuine post-easing shift toward midface injuries exists, this cohort is not large enough to demonstrate it, and the question is better addressed prospectively.

The multinomial analysis adds something a binary model restricted to isolated fractures cannot: combined mandibular-and-midface injuries behave differently from either isolated group, being associated with road-traffic, bicycle, and violence mechanisms and with one particular centre. Discarding them would mean discarding a clinically distinct and operationally demanding group.

**Micromobility.** Separating bicycle injuries from electric-scooter injuries changed the interpretation appreciably. Bicycle injuries rose during the restrictions and then fell back toward baseline, consistent with a temporary substitution of cycling for other activities. Electric-scooter injuries followed a continuous upward trend across the whole six years, with no level shifts at either interruption and a deceleration in the slope during restrictions. The growth of scooter-related facial injuries in this cohort is therefore best understood as part of the international secular rise that had accompanied the spread of shared micromobility schemes [10,11,27,28], rather than as a consequence of the pandemic. Pooling the two mechanisms into a single micromobility indicator conceals this. While prevention implications remain unchanged in substance, they are different in framing: measures such as helmet promotion, sobriety enforcement, speed limits, and infrastructure segregation address a growing and pandemic-independent source of facial injuries. We note that our data speak to the burden of these injuries rather than to the effectiveness of any specific countermeasure. The studies cited above are descriptive epidemiological series: they document the injury burdens and the risk-factor profiles that motivated such measures—helmets are worn by only a small minority of injured riders, and alcohol is frequently involved [27,28]—but none of them, and not the present study, evaluates whether any particular countermeasure reduces injuries. Effectiveness evidence would require a different design and lies outside the scope of this work.

**Demography and places of injury.** Our findings qualify the common account of a pandemic “shift to domestic injuries”: because the monthly rate of home-based falls was broadly constant, the apparent domestic shift is largely attributable to the temporary removal of street-based mechanisms. The growth in the oldest age band and falls in older women is real in rate terms, but it is gradual and not tied to either interruption in the segmented models; expressing it as a proportion of the relevant stratum, rather than as an absolute count across periods of unequal size, reduces it from an apparent near-doubling to a more moderate increase. It nonetheless supports continued fall prevention independent of pandemic status.

**Management.** The shift toward conservative management is the most robust compositional finding in this study. It appears as a significant level change at the easing in the time-series model, and it persists after adjustment for the fracture site, number of fracture sites, mechanism, associated injury, sex, age, and centre. That said, the very large centre effects in the same model—an approximately twenty-fold difference in the adjusted odds of conservative management between the extremes—indicate that local operative threshold is a far stronger determinant of management than period is, and any service-level inference must accommodate that.

### Strengths and Limitations

Strengths include the multicentre sample, the six-year window spanning clearly defined policy periods, the use of segmented regression with formal model selection for seasonality, explicit diagnostics for overdispersion and autocorrelation, centre-stratified and panel analyses, simulation-based uncertainty for the counterfactual, and patient-level multivariable modelling of compositional outcomes.

The limitations are substantial and constrain interpretation. **First and most importantly, the design is observational and uncontrolled.** There is no non-exposed comparison series, so temporal association cannot establish causation; over the same six years, the availability of shared e-scooters, the age structure of the population, patient routing, admission thresholds, and treatment preferences all changed independently of COVID-19 policies. **Second**, the primary outcome is hospital case volume, not incidence: no catchment denominator or person-time offset was available, so we cannot distinguish a change in injury occurrence from a change in the proportion of injuries reaching these three centres. **Third**, the numbers screened and excluded before cohort entry were not enumerated during data collection and cannot now be recovered, so the flow diagram begins at cohort entry rather than at screening. We therefore cannot demonstrate that case ascertainment was uniform across centres and periods, and selection bias at the point of record identification cannot be formally excluded. Two considerations bear on how much weight this should carry. Ascertainment was governed by a fixed diagnostic criterion—confirmed facial fracture—applied uniformly at all three centres throughout the study window, rather than by any judgement that could plausibly have shifted with pandemic phase; and the analyses that carry our principal conclusions are internal comparisons of composition and centre-stratified time-series models, which are less vulnerable to a constant ascertainment fraction than absolute volume estimates would be. Nevertheless, undetected drift in ascertainment remains a possible contributor to the volume findings, and this reinforces our decision not to interpret the pooled post-easing rise as a national rebound. **Fourth**, the contributing centres are heterogeneous, their shares change across periods, and the pooled volume finding depends on one of them. Although we can document that all three operated continuously with unchanged admission criteria, we cannot exclude undocumented drift in referral behaviour or record capture, and the pre-pandemic decline at Kosice suggests such drift occurred. **Fifth**, the interruption points are approximations of a staged policy process, and injury dates were available only to the month, so within-month exposure could not be apportioned. **Sixth**, age was recorded only in bands and was approximated by midpoints in continuous models. **Seventh**, mechanisms required a primary-cause rule for 5.6% of records; although results were stable under an alternative hierarchy, the rule is a judgement. **Eighth**, alcohol or substance involvement was undetermined in about 80% of records. **Ninth**, follow-up was not standardised, the early-outcome measure has a short horizon, and complication ascertainment could not be verified; hence, the outcome data support no strong claims about the quality of care. **Tenth**, some categories are sparse (e.g., 18 pre-restriction electric-scooter cases), and no strong inference rests on them. **Finally**, as a single-country study of three academic centres, absolute rates—particularly violence- and alcohol-related patterns—may not generalise, although internal period contrasts are less likely to be region-specific.

## 5. Conclusions

In this multicentre Slovak cohort, the periods of COVID-19 restrictions and subsequent easing were accompanied by measurable changes in the composition of maxillofacial trauma. The most consistent findings were a transient restriction-period shift toward mandibular and combined fractures, with a corresponding fall in the non-mandibular share; a temporary narrowing of the male predominance; and a sustained move toward conservative management that persisted after adjustment for case mix and treating centre. The increase in monthly case volume after the easing was confined to one of the three centres and was not robust to centre-stratified or panel analysis; it should not be read as a national rebound, and the associated counterfactual excess is too imprecise and too dependent on pre-trend extrapolation to support service planning on its own. The rise in electric-scooter facial injuries followed a continuous secular trend unrelated to either policy interruption, representing a growing prevention target in its own right. Taken together, these results describe period-associated patterns in the structure of facial trauma rather than demonstrating effects of the pandemic, and they indicate that multicentre time-series studies of trauma should model centre explicitly before drawing system-level conclusions.

## Figures and Tables

**Table 1:** Demographic and clinical characteristics by period. Values are *n* (%) within the period.

Characteristic	Pre-Restriction (*n* = 1361)	Restriction (*n* = 974)	Post-Easing (*n* = 1619)	Total (*n* = 3954)
Age 10–17 y	58 (4.3)	43 (4.4)	63 (3.9)	164 (4.1)
Age 18–29 y	362 (26.6)	239 (24.5)	387 (23.9)	988 (25.0)
Age 30–39 y	285 (20.9)	209 (21.5)	334 (20.6)	828 (20.9)
Age 40–49 y	226 (16.6)	165 (16.9)	288 (17.8)	679 (17.2)
Age 50–59 y	161 (11.8)	125 (12.8)	183 (11.3)	469 (11.9)
Age 60–69 y	140 (10.3)	101 (10.4)	164 (10.1)	405 (10.2)
Age ≥ 70 y	129 (9.5)	92 (9.4)	200 (12.4)	421 (10.6)
Age ≥ 60 y	269 (19.8)	193 (19.8)	364 (22.5)	826 (20.9)
Approx. mean age, y (SD)	42.0 (17.9)	42.4 (17.8)	43.5 (18.4)	42.7 (18.1)
Male	1061 (78.0)	715 (73.4)	1214 (75.0)	2990 (75.6)
Female	300 (22.0)	259 (26.6)	405 (25.0)	964 (24.4)
Alcohol/substance documented	275 (20.2)	165 (16.9)	368 (22.7)	808 (20.4)
Associated injury	557 (40.9)	372 (38.2)	626 (38.7)	1555 (39.3)
Tracheostomy	27 (2.0)	13 (1.3)	16 (1.0)	56 (1.4)

Age was recorded in bands; the approximate mean used band midpoints (Kruskal–Wallis, *p* = 0.078). Sex distribution across periods, *p* = 0.031.

**Table 2:** Patient distribution by treating centre and period.

Centre	Pre-Restriction	Restriction	Post-Easing	Total
Bratislava	669 (49.2)	498 (51.1)	916 (56.6)	2083 (52.7)
Martin	453 (33.3)	359 (36.9)	506 (31.3)	1318 (33.3)
Kosice	239 (17.6)	117 (12.0)	197 (12.2)	553 (14.0)
Total	1361	974	1619	3954
Mean cases/month, Bratislava	25.7	22.6	38.2	—
Mean cases/month, Martin	17.4	16.3	21.1	—
Mean cases/month, Kosice	9.2	5.3	8.2	—

Values are *n* (%) within the period. Periods comprise 26, 22, and 24 months, respectively.

**Table 3:** Principal segmented negative binomial model of the monthly count of treated cases (72 months).

Term	Count Ratio (95% CI)	*p*
Underlying trend (per month)	0.992 (0.982–1.001)	0.077
Level changes, March 2020 restrictions	0.84 (0.67–1.04)	0.111
Slope changes after restrictions (per month)	1.014 (0.998–1.030)	0.083
Level changes, January 2022 easing	1.50 (1.22–1.86)	<0.001
Slope changes after easing (per month)	0.995 (0.979–1.010)	0.502
Seasonality (sine, 12 months)	0.833 (0.784–0.884)	<0.001
Seasonality (cosine, 12 months)	0.814 (0.768–0.863)	<0.001

Negative binomial (NB2), α = 0.0119. Pearson’s dispersion of the corresponding Poisson model: 1.76. Full coefficient sets for all sensitivity models are in Table S1.

**Table 4:** Between-centre heterogeneity in the level changes at each interruption.

Analysis	Restriction Level Changes (95% CI)	Easing Level Changes (95% CI)
Pooled series (principal model)	0.84 (0.67–1.04)	1.50 (1.22–1.86)
Centre–month panel, centre fixed effects, cluster-robust	0.84 (0.71–0.99)	1.40 (0.92–2.14)
Bratislava only (*n* = 2083)	0.77 (0.58–1.02)	1.86 (1.42–2.43)
Martin only (*n* = 1318)	0.98 (0.73–1.32)	1.32 (0.99–1.75)
Kosice only (*n* = 553)	0.64 (0.32–1.28)	0.80 (0.42–1.50)
Leave-one-out: excluding Bratislava	0.92 (—)	1.15 (0.88–1.50)
Leave-one-out: excluding Martin	0.77 (—)	1.59 (1.21–2.09)
Leave-one-out: excluding Kosice	0.85 (—)	1.64 (1.34–2.01)

Likelihood-ratio test of centre × interruption interaction: χ^2^ = 27.28, df = 4, *p* < 0.001. — denotes that a 95% confidence interval is not reported; leave-one-out estimates for the restriction interruption are presented as point estimates only.

**Table 5:** Fracture sites by periods.

Fracture Site	Pre-Restriction	Restriction	Post-Easing
Non-mandibular (midface/upper facial third)	905 (66.5)	594 (61.0)	1137 (70.2)
Mandible	369 (27.1)	301 (30.9)	394 (24.3)
Combined	86 (6.3)	76 (7.8)	86 (5.3)
Not classifiable	1 (0.1)	3 (0.3)	2 (0.1)
Non-mandibular: mandibular ratio	2.45	1.97	2.89

Values are *n* (%) within the period. Distribution across periods, *p* < 0.001 (*q* < 0.001).

**Table 6:** Multinomial logistic regression of fracture sites (*n* = 3948). Reference outcome: non-mandibular fracture; reference period: pre-restriction; reference mechanism: fall; reference centre: Bratislava.

Variable	Mandible RRR (95% CI)	*p*	Combined RRR (95% CI)	*p*
Restriction period	1.25 (1.03–1.51)	0.023	1.41 (1.01–1.97)	0.042
Post-easing period	0.85 (0.72–1.01)	0.068	0.85 (0.62–1.17)	0.331
Interpersonal violence	0.95 (0.77–1.16)	0.586	1.66 (1.11–2.50)	0.014
Bicycle	0.61 (0.47–0.78)	<0.001	1.76 (1.12–2.76)	0.014
Electric scooter	0.83 (0.56–1.22)	0.344	1.83 (0.90–3.73)	0.097
Road traffic	0.49 (0.35–0.68)	<0.001	2.85 (1.78–4.57)	<0.001
Sport	0.44 (0.31–0.64)	<0.001	0.83 (0.39–1.78)	0.637
Other	0.64 (0.45–0.90)	0.010	2.16 (1.27–3.66)	0.004
Male sex	0.86 (0.71–1.04)	0.111	1.12 (0.79–1.59)	0.514
Age (per 10 years)	0.71 (0.67–0.75)	<0.001	0.96 (0.87–1.06)	0.427
Age squared (per 10 years)	1.03 (1.01–1.06)	0.012	0.95 (0.91–1.00)	0.046
Centre: Martin	0.90 (0.77–1.06)	0.214	0.83 (0.60–1.14)	0.247
Centre: Kosice	0.54 (0.42–0.69)	<0.001	1.85 (1.33–2.57)	<0.001

RRR, relative risk ratio. An RRR < 1 for the mandible outcome indicates greater relative association with a non-mandibular fracture.

**Table 7:** Injury mechanisms by periods, with the primary-cause rule of Section 2.4 applied.

Mechanism	Pre-Restriction	Restriction	Post-Easing
Fall	465 (34.2)	329 (33.8)	556 (34.3)
Interpersonal violence	436 (32.0)	262 (26.9)	447 (27.6)
Bicycle	156 (11.5)	162 (16.6)	204 (12.6)
Road traffic	120 (8.8)	77 (7.9)	104 (6.4)
Electric scooter	18 (1.3)	37 (3.8)	100 (6.2)
Sport	83 (6.1)	41 (4.2)	100 (6.2)
Other	83 (6.1)	66 (6.8)	108 (6.7)
*Micromobility (bicycle + scooter), supplementary*	*174 (12.8)*	*199 (20.4)*	*304 (18.8)*

Values are *n* (%) within the period. Distribution across periods, *p* < 0.001 (*q* < 0.001). Supplementary composite row Micromobility (bicycle + electric scooter) duplicates counts reported above and is excluded from column totals and from the significance test. Segmented binomial models of the monthly share are reported in Table S5.

**Table 8:** Places of injury by periods.

Location	Pre-Restriction	Restriction	Post-Easing
Urban street	680 (50.0)	471 (48.4)	889 (54.9)
Home	281 (20.6)	205 (21.0)	355 (21.9)
Sports facility	84 (6.2)	54 (5.5)	113 (7.0)
Restaurant/bar	68 (5.0)	50 (5.1)	68 (4.2)
Workplace	59 (4.3)	41 (4.2)	57 (3.5)
Cultural venue	30 (2.2)	17 (1.7)	24 (1.5)
Hospital	10 (0.7)	10 (1.0)	13 (0.8)
Public transport	11 (0.8)	6 (0.6)	11 (0.7)
Other	138 (10.1)	120 (12.3)	89 (5.5)

Values are *n* (%) within period. Distribution across periods, *p* < 0.001 (*q* < 0.001).

**Table 9:** Management modality, timing of definitive care, and early outcome by periods.

Variable	Pre-Restriction	Restriction	Post-Easing
Conservative	594 (43.6)	461 (47.3)	865 (53.4)
Closed reduction	81 (6.0)	53 (5.4)	84 (5.2)
Open reduction and internal fixation	686 (50.4)	460 (47.2)	670 (41.4)
Definitive care ≤ 24 h	254 (18.7)	127 (13.0)	363 (22.4)
Definitive care > 96 h	524 (38.5)	374 (38.4)	595 (36.8)
Uncomplicated early healing	1279 (94.0)	926 (95.1)	1526 (94.3)
Long-term sequelae documented	306 (22.5)	245 (25.2)	324 (20.0)

Values are *n* (%) within the period. Management across periods, *p* < 0.001; timing, *p* < 0.001; uncomplicated early healing, *p* = 0.51; sequelae, *p* = 0.009.

## Data Availability

The anonymised minimal dataset supporting the conclusions of this article is included as Supplementary Materials. Further inquiries can be directed to the corresponding author.

## Supplementary Materials

The following supporting information can be downloaded at https://www.mdpi.com/article/10.3390/medicina62091628/s1. File S1: STROBE Statement—checklist of items for reports of observational studies. Table S1: Complete coefficient sets for the principal and all sensitivity time-series models. Table S2: Period comparisons before and after false-discovery-rate correction. Table S3: Variable-level missing data by centre and period. Table S4: Seasonality specification comparison. Table S5: Segmented binomial models of monthly proportions for all structural outcomes. Figure S1: Residual diagnostics for the principal segmented negative-binomial model: (a) deviance residuals over time, (b) autocorrelation function, (c) partial autocorrelation function. Dashed lines are 95% bounds. Figure S2: Flow diagram of the study cohort. Because records were transcribed only for the study diagnoses during manual case identification, presentations not meeting those diagnoses were never enumerated, and counts of screened and excluded records are not retrievable (Section 2.2, Strengths and Limitations Section). The diagram therefore begins at cohort entry and documents the onward flow into each analysis. All 3954 patients contribute to the period-comparative and centre-stratified analyses; the six records whose fracture region could not be classified are omitted from the fracture-site models only.

## Abbreviations

aOR, adjusted odds ratio; CI, confidence interval; COVID-19, coronavirus disease 2019; CR, count ratio; HAC, heteroscedasticity- and autocorrelation-consistent; ITS, interrupted time-series; NB2, negative binomial (quadratic variance); ORIF, open reduction and internal fixation; RRR, relative risk ratio; STROBE, Strengthening the Reporting of Observational Studies in Epidemiology.
