# Supplementary material for: Volume and Structure of Maxillofacial Trauma Before, During, and After COVID-19 Restrictions: A Multicentre Interrupted Time-Series and Period-Comparative Analysis of 3954 Patients
Source: Medicina (Kaunas). 2026 Aug 25;62(9):1628. doi: 10.3390/medicina62091628 (PMC13609789; doi:10.3390/medicina62091628)
Supplement: Supplementary file 1 [file medicina-62-01628-s001.zip › Supplementary Materials-Figures.pdf]

Supplementary Figures

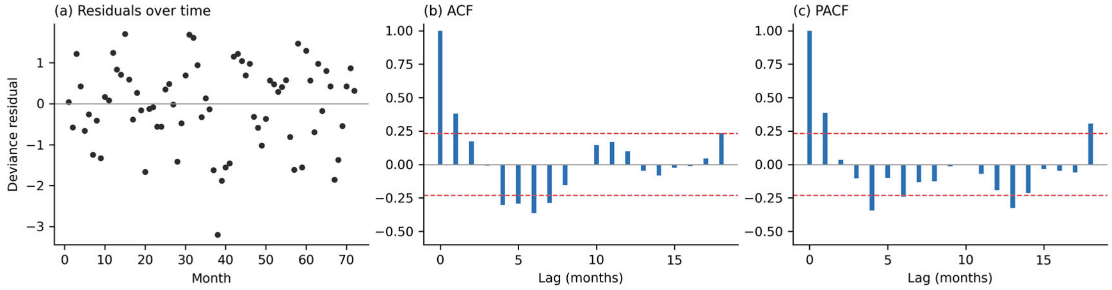

Figure S1. Residual diagnostics for the principal segmented negative-binomial model: (a) deviance residuals over time, (b) autocorrelation function, (c) partial autocorrelation function. Dashed lines are 95% bounds.

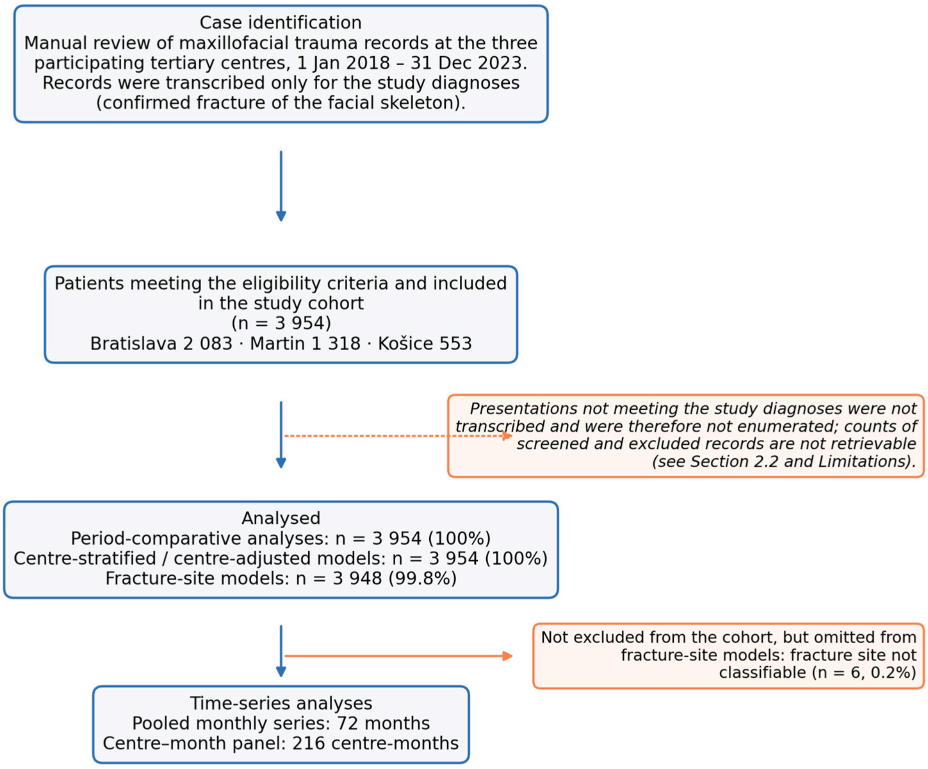

Figure S2. Flow diagram of the study cohort. Because records were transcribed only for the study diagnoses during manual case identification, presentations not meeting those diagnoses were never enumerated, and counts of screened and excluded records are not retrievable (Section 2.2, Section 4.1). The diagram therefore begins at cohort entry and documents the onward flow into each analysis. All 3954 patients contribute to the period-comparative and centre-stratified analyses; the six records whose fracture region could not be classified are omitted from the fracture-site models only.
