# Supplementary material for: Volume and Structure of Maxillofacial Trauma Before, During, and After COVID-19 Restrictions: A Multicentre Interrupted Time-Series and Period-Comparative Analysis of 3954 Patients
Source: Medicina (Kaunas). 2026 Aug 25;62(9):1628. doi: 10.3390/medicina62091628 (PMC13609789; doi:10.3390/medicina62091628)
Supplement: Supplementary file 1 [file medicina-62-01628-s001.zip › Supplementary Materials-File S1.pdf]

Supplementary Materials

STROBE Statement

Manuscript ID: medicina-4477376

Title: Volume and Structure of Maxillofacial Trauma Before, During, and After COVID-19 Restrictions: A Multicentre Interrupted Time-Series and Period-Comparative Analysis of 3954 Patients

File S1. STROBE Statement — checklist of items for reports of observational studies

Item numbers follow the STROBE statement for cohort studies (von Elm et al., 2007). Page and section references are to the revised manuscript.

No.	Recommendation	Reported in
1a	Indicate the study design with a commonly used term in the title or abstract	Title; Abstract
1b	Provide in the abstract an informative and balanced summary of what was done and what was found	Abstract
2	Explain the scientific background and rationale for the investigation	Section 1
3	State specific objectives, including any prespecified hypotheses	Section 1.1
4	Present key elements of study design early in the paper	Section 2.1
5	Describe the setting, locations, and relevant dates, including periods of recruitment, exposure, follow-up, and data collection	Section 2.1; Section 2.3
6a	Give the eligibility criteria, and the sources and methods of selection of participants	Section 2.2
6b	For matched studies, give matching criteria and number of exposed and unexposed	Not applicable
7	Clearly define all outcomes, exposures, predictors, potential	Section 2.3; Section 2.4

No.	Recommendation	Reported in
	confounders, and effect modifiers	
8	For each variable of interest, give sources of data and details of methods of assessment	Section 2.1; Section 2.4
9	Describe any efforts to address potential sources of bias	Section 2.6; Section 4.1
10	Explain how the study size was arrived at	Section 2.2 (all eligible patients in the study window)
11	Explain how quantitative variables were handled in the analyses	Section 2.4 (age bands, midpoints, continuous and quadratic terms)
12a	Describe all statistical methods, including those used to control for confounding	Section 2.6
12b	Describe any methods used to examine subgroups and interactions	Section 2.6 (centre-stratified models; centre × interruption; period × mechanism)
12c	Explain how missing data were addressed	Section 2.5; Table S3
12d	If applicable, explain how loss to follow-up was addressed	Section 2.4; Section 4.1 (follow-up not standardised)
12e	Describe any sensitivity analyses	Section 2.6; Section 3.2; Section 3.3; Tables S1, S4
**13a**	**Report the numbers of individuals at each stage of the study**	**Section 3.1; Figure S2. All 3954 included patients are accounted for at every analysis stage, and centre is complete. Note: numbers screened and excluded *before* cohort entry are not retrievable. Records were transcribed during manual case identification only where the study diagnoses were met, and non-qualifying presentations were never enumerated. The flow diagram therefore begins at cohort entry (n = 3954). See Section 2.2 and Section 4.1 (third limitation).**
13b	Give reasons for non-participation at each stage	Not retrievable; see item 13a and Section 2.2
13c	Consider use of a flow diagram	Figure S2
14a	Give characteristics of study participants and information on exposures and potential confounders	Table 1; Table 2

No.	Recommendation	Reported in
14b	Indicate number of participants with missing data for each variable of interest	Section 2.5; Table S3
14c	Summarise follow-up time	Section 2.4 (early outcome assessed to first post-treatment review, within six weeks)
15	Report numbers of outcome events or summary measures over time	Section 3.1; Section 3.2; Figure 1
16a	Give unadjusted estimates and, if applicable, confounder-adjusted estimates and their precision	Tables 3, 4, 6; Section 3.7; Tables S1, S5
16b	Report category boundaries when continuous variables were categorised	Section 2.4 (age bands; ≥60 and ≥70 thresholds)
16c	If relevant, consider translating estimates of relative risk into absolute risk	Section 3.1; Tables 1, 5, 7, 8, 9 (within-period percentages and monthly rates)
17	Report other analyses done	Section 2.6; Section 3.2–3.7; Tables S1, S2, S4, S5
18	Summarise key results with reference to study objectives	Section 4 (opening); Section 5
19	Discuss limitations, taking into account sources of potential bias or imprecision	Section 4.1
20	Give a cautious overall interpretation of results	Section 4; Section 5
21	Discuss the generalisability of the study results	Section 4.1 (final limitation)
22	Give the source of funding and the role of the funders	Funding statement

An explanation and elaboration article discusses each checklist item and gives methodological background and published examples of transparent reporting. The STROBE checklist is best used in conjunction with that article, freely available on the websites of PLoS Medicine, Annals of Internal Medicine, and Epidemiology.
