# Supplementary material for: Volume and Structure of Maxillofacial Trauma Before, During, and After COVID-19 Restrictions: A Multicentre Interrupted Time-Series and Period-Comparative Analysis of 3954 Patients
Source: Medicina (Kaunas). 2026 Aug 25;62(9):1628. doi: 10.3390/medicina62091628 (PMC13609789; doi:10.3390/medicina62091628)
Supplement: Supplementary file 1 [file medicina-62-01628-s001.zip › Supplementary Materials-Tables.pdf]

Supplementary Materials

Manuscript ID: medicina-4477376

Title: Volume and Structure of Maxillofacial Trauma Before, During, and After COVID-19 Restrictions: A Multicentre Interrupted Time-Series and Period-Comparative Analysis of 3954 Patients

Table S1. Complete coefficient sets for the principal and all sensitivity time-series models

All estimates are count ratios (ratios of expected monthly counts) with 95% confidence intervals. The outcome is the monthly number of treated cases (72 months).

S1a. Principal model: segmented negative binomial (NB2), α = 0.0119

Term	Count ratio	95% CI	*p*
Intercept	59.002	51.206–67.983	<0.001
Underlying trend (per month)	0.992	0.982–1.001	0.077
Level change, Mar 2020	0.837	0.672–1.042	0.111
Slope change after restrictions	1.014	0.998–1.030	0.083
Level change, Jan 2022	1.503	1.216–1.857	<0.001
Slope change after easing	0.995	0.979–1.010	0.502
Seasonality (sine)	0.832	0.784–0.884	<0.001
Seasonality (cosine)	0.814	0.768–0.863	<0.001

Pearson dispersion statistic of the corresponding Poisson model: 1.758.

S1b. Poisson with HC1 robust standard errors

Term	Count ratio	95% CI	*p*
Intercept	58.434	52.817–64.648	<0.001
Underlying trend (per month)	0.992	0.986–0.998	0.010
Level change, Mar 2020	0.848	0.691–1.041	0.114
Slope change after restrictions	1.014	1.000–1.028	0.052
Level change, Jan 2022	1.482	1.195–1.839	<0.001
Slope change after easing	0.994	0.980–1.008	0.415
Seasonality (sine)	0.841	0.788–0.896	<0.001
Seasonality (cosine)	0.817	0.775–0.862	<0.001

S1c. Negative binomial with Newey–West HAC(6) standard errors

Term	Count ratio	95% CI	*p*
Intercept	59.002	53.469–65.107	<0.001
Underlying trend (per month)	0.992	0.986–0.997	0.005
Level change, Mar 2020	0.837	0.671–1.044	0.115
Slope change after restrictions	1.014	0.999–1.029	0.070
Level change, Jan 2022	1.503	1.185–1.906	<0.001

Term	Count ratio	95% CI	*p*
Slope change after easing	0.995	0.981–1.008	0.443
Seasonality (sine)	0.832	0.751–0.923	<0.001
Seasonality (cosine)	0.814	0.770–0.861	<0.001

S1d. Negative binomial including the previous month's log-count

Term	Count ratio	95% CI	*p*
Intercept	14.067	6.201–31.908	<0.001
Underlying trend (per month)	0.995	0.985–1.004	0.275
Level change, Mar 2020	0.891	0.723–1.100	0.283
Slope change after restrictions	1.009	0.994–1.024	0.223
Level change, Jan 2022	1.317	1.066–1.627	0.011
Slope change after easing	0.995	0.980–1.009	0.471
Seasonality (sine)	0.915	0.848–0.988	0.023
Seasonality (cosine)	0.844	0.796–0.895	<0.001
Log count, previous month	1.420	1.165–1.730	<0.001

S1e. Generalised least squares with AR(1) error on the log count (ϱ = 0.440)

Term	Count ratio	95% CI	*p*
Intercept	58.540	43.383–78.992	<0.001
Underlying trend (per month)	0.992	0.974–1.010	0.400
Level change, Mar 2020	0.776	0.561–1.073	0.130
Slope change after restrictions	1.014	0.986–1.042	0.325
Level change, Jan 2022	1.521	1.103–2.097	0.013
Slope change after easing	0.995	0.969–1.023	0.741
Seasonality (sine)	0.815	0.741–0.898	<0.001
Seasonality (cosine)	0.805	0.731–0.887	<0.001

S1f. Negative binomial with two harmonic pairs

Term	Count ratio	95% CI	*p*
Intercept	59.217	51.331–68.315	<0.001
Underlying trend (per month)	0.991	0.982–1.001	0.073
Level change, Mar 2020	0.840	0.673–1.047	0.121
Slope change after restrictions	1.014	0.998–1.030	0.087
Level change, Jan 2022	1.519	1.226–1.883	<0.001
Slope change after easing	0.994	0.979–1.010	0.486
Seasonality (sine)	0.830	0.781–0.882	<0.001
Seasonality (cosine)	0.815	0.768–0.864	<0.001
Seasonality (sine, 2nd harmonic)	0.984	0.929–1.043	0.598
Seasonality (cosine, 2nd harmonic)	1.018	0.961–1.079	0.546

Model S1d removed residual autocorrelation (Ljung–Box p = 0.144 at lag 12). As explained in Section 2.6, this dynamic specification conditions on an intermediate variable; its interruption coefficients are attenuated and are interpreted as a lower bound.

S1g. Residual diagnostics for the principal model

Diagnostic	Value
Durbin–Watson statistic	1.236
Ljung–Box test, lag 6	$\chi^2 = 37.76$, p <0.001
Ljung–Box test, lag 12	$\chi^2 = 51.66$, p <0.001
Ljung–Box test, lag 18	$\chi^2 = 58.22$, p <0.001
Residual ACF at lag 1	0.380 (95% bound ±0.231)
Residual PACF at lag 1	0.385

See Figure S1 for residual plots, ACF, and PACF.

S1h. Breakpoint sensitivity: grid of 25 models shifting each interruption by up to ±2 months

Quantity	Range across the 25 models
Level change at restrictions	0.806 to 0.969
Significant at p < 0.05 (restrictions)	0 of 25 models (minimum p = 0.051)
Level change at easing	1.299 to 1.560
Significant at p < 0.05 (easing)	25 of 25 models (maximum p = 0.020)

S1i. Cumulative excess relative to the modelled counterfactual

Period	Observed	Counterfactual (95% CI)	Excess % (95% CI)
Restriction (months 27–48)	974	985 (782–1246)	-1.1 (-21.9 to 24.5)
Post-easing (months 49–72)	1619	863 (556–1375)	87.7 (17.7 to 191.2)

Sensitivity of the post-easing excess to model specification

Specification	Counterfactual total	Excess %
Principal model (one harmonic pair)	863	87.7
Two harmonic pairs	856	89.2
No seasonality term	834	94.2
Pre-interruption trend not extrapolated	1290	25.5

Intervals are 95% confidence intervals for the expected (mean) count, obtained from 10 000 Monte Carlo draws of the coefficient vector. They propagate the sampling uncertainty of the model coefficients only and do not incorporate the dispersion of the negative-binomial observation process; they are therefore not prediction intervals for an observed count, which would be wider. The excess is computed as 100 × (ΣY_obs − ΣY_cf)/ΣY_cf.

Table S2. Period comparisons before and after false-discovery-rate correction

Benjamini–Hochberg procedure applied across the family of secondary period comparisons.

Comparison across periods	*p*	*q* (FDR)	Significant at q < 0.05
Injury mechanism	<0.001	<0.001	Yes
Time to definitive care	<0.001	<0.001	Yes
Same-day (≤24 h) definitive care	<0.001	<0.001	Yes
Micromobility injury (composite)	<0.001	<0.001	Yes
Management modality	<0.001	<0.001	Yes

Comparison across periods	*p*	*q* (FDR)	Significant at q < 0.05
Place of injury	<0.001	<0.001	Yes
Fracture site	<0.001	<0.001	Yes
Documented alcohol/substance involvement	0.002	0.004	Yes
Long-term sequelae	0.009	0.016	Yes
Age ≥70 years	0.015	0.024	Yes
Sex	0.031	0.045	Yes
Tracheostomy	0.070	0.094	No
Age ≥60 years	0.122	0.150	No
Associated injury	0.320	0.366	No
Age band distribution	0.422	0.450	No
Early complication	0.512	0.512	No

Table S3. Variable-level missing data by centre and period

Counts of missing values within the included cohort (n = 3954).

Variable	Missing, n (%)	Pre-restr.	Restr.	Post-eas.	BA	MT	KE
Treating centre	0 (0.0)	0	0	0	0	0	0
Year of injury	0 (0.0)	0	0	0	0	0	0
Month of injury	0 (0.0)	0	0	0	0	0	0
Sex	0 (0.0)	0	0	0	0	0	0
Age band	0 (0.0)	0	0	0	0	0	0
Injury mechanism	0 (0.0)	0	0	0	0	0	0
Place of injury	0 (0.0)	0	0	0	0	0	0
Alcohol or psychoactive-substance involvement	0 (0.0)	0	0	0	0	0	0
Time from injury to definitive care	0 (0.0)	0	0	0	0	0	0
Associated injury	0 (0.0)	0	0	0	0	0	0
Fracture site (fractured regions)	0 (0.0)	0	0	0	0	0	0
Antibiotic treatment †	0 (0.0)	0	0	0	0	0	0
Management modality	0 (0.0)	0	0	0	0	0	0
Osteosynthesis material used †	0 (0.0)	0	0	0	0	0	0
Early healing complication	0 (0.0)	0	0	0	0	0	0
Tracheostomy	0 (0.0)	0	0	0	0	0	0
Long-term sequelae after treatment	0 (0.0)	0	0	0	0	0	0
Operating surgeon's age band †	1908 (48.3)	586	460	862	1013	877	18

BA, Bratislava; MT, Martin; KE, Kosice. Variable names are given in English; the deposited minimal dataset records them in Slovak. † Variable present in the source dataset but not used in any analysis reported in this manuscript.

No analysis variable has missing values, so no patient is excluded from any analysis for missing data. The operating surgeon's age band is missing in 1908 records (48.3%) by design, because it is recorded only for operatively managed cases.

Informative non-missing category. Alcohol or psychoactive-substance involvement was recorded as "not determined" in 3146 records (79.6%), with comparable proportions in each period (pre-restriction 79.8%, restriction

83.1%, post-easing 77.3%). This is not missingness in the statistical sense but incomplete ascertainment, and alcohol-related findings are qualified accordingly in the manuscript.

Records not captured before cohort entry. This table concerns missing values within the included cohort. As set out in Section 2.2 and Section 4.1, presentations not meeting the study diagnoses were not transcribed during manual case identification and were never enumerated; counts of screened and excluded records are therefore not retrievable and cannot be tabulated here.

Table S4. Seasonality specification comparison

Segmented negative-binomial models of the monthly case count, differing only in the seasonality term.

Seasonality specification	Parameters	Log-likelihood	AIC	BIC
No seasonality term	6	-292.76	597.5	-207.6
One harmonic pair (sine + cosine, 12 months) **(selected)**	8	-262.99	542.0	-200.6
Two harmonic pairs	10	-262.69	545.4	-193.5
Three harmonic pairs	12	-261.87	547.7	-184.8
11 calendar-month indicators	17	-258.38	550.8	-165.2

The single harmonic pair minimised AIC and was retained for the principal model.

Table S5. Segmented binomial models of monthly proportions for all structural outcomes

Each outcome is the monthly proportion of the stated characteristic among treated cases, modelled with the same time, level, slope, and harmonic-seasonality structure as the principal model, with HC1 standard errors. Estimates are odds ratios with 95% confidence intervals. All outcomes examined are reported, whether or not significant.

Non-mandibular (midface/upper facial third) fracture

Term	Odds ratio	95% CI	*p*
Trend	1.006	0.995–1.018	0.277
Level, Mar 2020	0.541	0.381–0.767	<0.001
Slope change, restrictions	1.023	0.998–1.048	0.068
Level, Jan 2022	0.852	0.615–1.181	0.337
Slope change, easing	0.990	0.966–1.016	0.453

Electric-scooter injury

Term	Odds ratio	95% CI	*p*
Trend	1.134	1.052–1.221	<0.001
Level, Mar 2020	0.636	0.267–1.512	0.305
Slope change, restrictions	0.884	0.812–0.961	0.004
Level, Jan 2022	1.715	0.871–3.378	0.119
Slope change, easing	1.006	0.958–1.056	0.811

Bicycle injury

Term	Odds ratio	95% CI	p
Trend	1.019	0.992–1.048	0.172
Level, Mar 2020	1.106	0.692–1.768	0.675
Slope change, restrictions	0.972	0.938–1.007	0.113
Level, Jan 2022	0.795	0.497–1.270	0.337
Slope change, easing	1.021	0.988–1.055	0.215

Micromobility (bicycle + scooter, composite)

Term	Odds ratio	95% CI	p
Trend	1.031	1.004–1.060	0.026
Level, Mar 2020	1.064	0.708–1.600	0.764
Slope change, restrictions	0.962	0.932–0.993	0.017
Level, Jan 2022	0.986	0.650–1.496	0.947
Slope change, easing	1.020	0.993–1.047	0.156

Interpersonal violence

Term	Odds ratio	95% CI	p
Trend	0.987	0.972–1.002	0.085
Level, Mar 2020	1.032	0.685–1.555	0.880
Slope change, restrictions	1.007	0.985–1.030	0.541
Level, Jan 2022	1.186	0.841–1.672	0.330
Slope change, easing	0.999	0.975–1.024	0.953

Road-traffic injury

Term	Odds ratio	95% CI	p
Trend	1.017	0.992–1.041	0.179
Level, Mar 2020	0.564	0.302–1.052	0.072
Slope change, restrictions	1.005	0.956–1.057	0.832
Level, Jan 2022	0.665	0.353–1.252	0.206
Slope change, easing	0.975	0.926–1.026	0.325

Sport injury

Term	Odds ratio	95% CI	p
Trend	0.981	0.955–1.007	0.145
Level, Mar 2020	0.951	0.428–2.111	0.901
Slope change, restrictions	1.017	0.959–1.078	0.582
Level, Jan 2022	1.480	0.649–3.378	0.352
Slope change, easing	1.005	0.943–1.071	0.879

Age ≥60 years

Term	Odds ratio	95% CI	p

Term	Odds ratio	95% CI	p
Trend	1.013	0.997–1.031	0.121
Level, Mar 2020	0.777	0.539–1.119	0.175
Slope change, restrictions	0.997	0.972–1.023	0.836
Level, Jan 2022	0.809	0.567–1.154	0.242
Slope change, easing	1.008	0.981–1.035	0.572

Age ≥70 years

Term	Odds ratio	95% CI	p
Trend	1.015	0.998–1.032	0.092
Level, Mar 2020	0.861	0.541–1.372	0.530
Slope change, restrictions	0.985	0.952–1.020	0.397
Level, Jan 2022	0.906	0.515–1.593	0.731
Slope change, easing	1.029	0.989–1.070	0.163

Female sex

Term	Odds ratio	95% CI	p
Trend	0.996	0.982–1.010	0.599
Level, Mar 2020	1.531	1.078–2.174	0.017
Slope change, restrictions	0.993	0.968–1.018	0.566
Level, Jan 2022	1.152	0.825–1.608	0.406
Slope change, easing	1.002	0.977–1.028	0.847

Conservative management

Term	Odds ratio	95% CI	p
Trend	1.023	1.003–1.042	0.023
Level, Mar 2020	0.875	0.605–1.265	0.478
Slope change, restrictions	0.977	0.952–1.003	0.086
Level, Jan 2022	1.559	1.117–2.176	0.009
Slope change, easing	0.986	0.964–1.009	0.222

Open reduction and internal fixation

Term	Odds ratio	95% CI	p
Trend	0.978	0.962–0.995	0.011
Level, Mar 2020	1.226	0.865–1.738	0.252
Slope change, restrictions	1.019	0.995–1.044	0.129
Level, Jan 2022	0.689	0.513–0.927	0.014
Slope change, easing	1.016	0.996–1.037	0.125

Same-day (≤24 h) definitive care

Term	Odds ratio	95% CI	p

Term	Odds ratio	95% CI	*p*
Trend	1.012	0.994–1.031	0.187
Level, Mar 2020	0.551	0.354–0.856	0.008
Slope change, restrictions	0.988	0.958–1.019	0.441
Level, Jan 2022	1.969	1.295–2.994	0.002
Slope change, easing	0.999	0.969–1.031	0.970

Documented alcohol/substance involvement

Term	Odds ratio	95% CI	*p*
Trend	0.985	0.966–1.004	0.115
Level, Mar 2020	0.957	0.622–1.474	0.843
Slope change, restrictions	1.019	0.986–1.053	0.270
Level, Jan 2022	1.220	0.805–1.849	0.349
Slope change, easing	1.008	0.974–1.043	0.655

Early healing complication

Term	Odds ratio	95% CI	*p*
Trend	0.964	0.935–0.993	0.017
Level, Mar 2020	1.506	0.753–3.010	0.247
Slope change, restrictions	1.031	0.982–1.082	0.218
Level, Jan 2022	1.058	0.574–1.949	0.858
Slope change, easing	1.019	0.975–1.064	0.410
